# Lifestyle interventions targeting adiposity in adults with psoriasis: a systematic review and network meta-analysis

**DOI:** 10.3389/fnut.2026.1912400

**Published:** 2026-08-06

**Authors:** Ran Huo, Wenhao Li, Haoran Luo, Ning Su, Ting Wang, Haicheng Gou, Shijie Zhou

**Affiliations:** 1 School of Physical Education and Training, Capital University of Physical Education and Sports, Beijing, China; 2 School of Physical Education, Northeastern University, Shenyang, Liaoning, China; 3 Institute of Physical Culture, Sports and Tourism, Peter the Great St. Petersburg Polytechnic University, Saint Petersburg, Russia; 4 Faculty of Health Sciences and Sports, Macao Polytechnic University, Macau, Macao SAR, China; 5 School of Competitive Sports, Beijing Sport University, Beijing, China; 6 School of Physical Education and Sports, Central China Normal University, Wuhan, China

**Keywords:** lifestyle intervention, low-energy diet, network meta-analysis, obesity, psoriasis, weight loss

## Abstract

**Objective:**

Excess adiposity drives inflammation and impairs treatment response in psoriasis. We compared the effectiveness of dietary energy restriction, aerobic exercise, and combined diet–exercise interventions in adults with psoriasis and overweight or obesity.

**Methods:**

We searched four English- and four Chinese-language databases from inception to 15 January 2026 for randomized controlled trials of low-energy diet (LED), aerobic exercise (AE), diet plus aerobic exercise (D+AE), or usual care (UC). Primary outcomes were body mass index (BMI), body weight, and waist circumference; secondary outcomes included Psoriasis Area and Severity Index (PASI). A Bayesian random-effects network meta-analysis was performed with UC as the reference; certainty of evidence was assessed with Confidence in Network Meta-Analysis (CINeMA).

**Results:**

Nine trials (1,066 participants) were included. Versus UC, LED produced the most consistent reductions in BMI (mean difference −2.89 kg/m^2^; 95% credible interval −4.78 to −0.73) and body weight (−7.97 kg; −13.07 to −0.81). The AE estimate was informed by only one trial and was highly uncertain. D+AE yielded the most favorable numerical PASI estimate (−2.48; −6.26 to 1.46), although the credible interval crossed the null. Certainty of evidence was low across outcomes.

**Conclusion:**

Low-energy diet showed the most consistent short-term reductions in BMI and body weight, whereas effects on waist circumference and PASI remained uncertain. Given the sparse network and low certainty of evidence, comparisons between lifestyle strategies should be regarded as hypothesis-generating rather than confirmatory.

**Systematic review registration:**

www.crd.york.ac.uk/prospero, CRD420261291403.

## Graphical abstract

**Figure:**
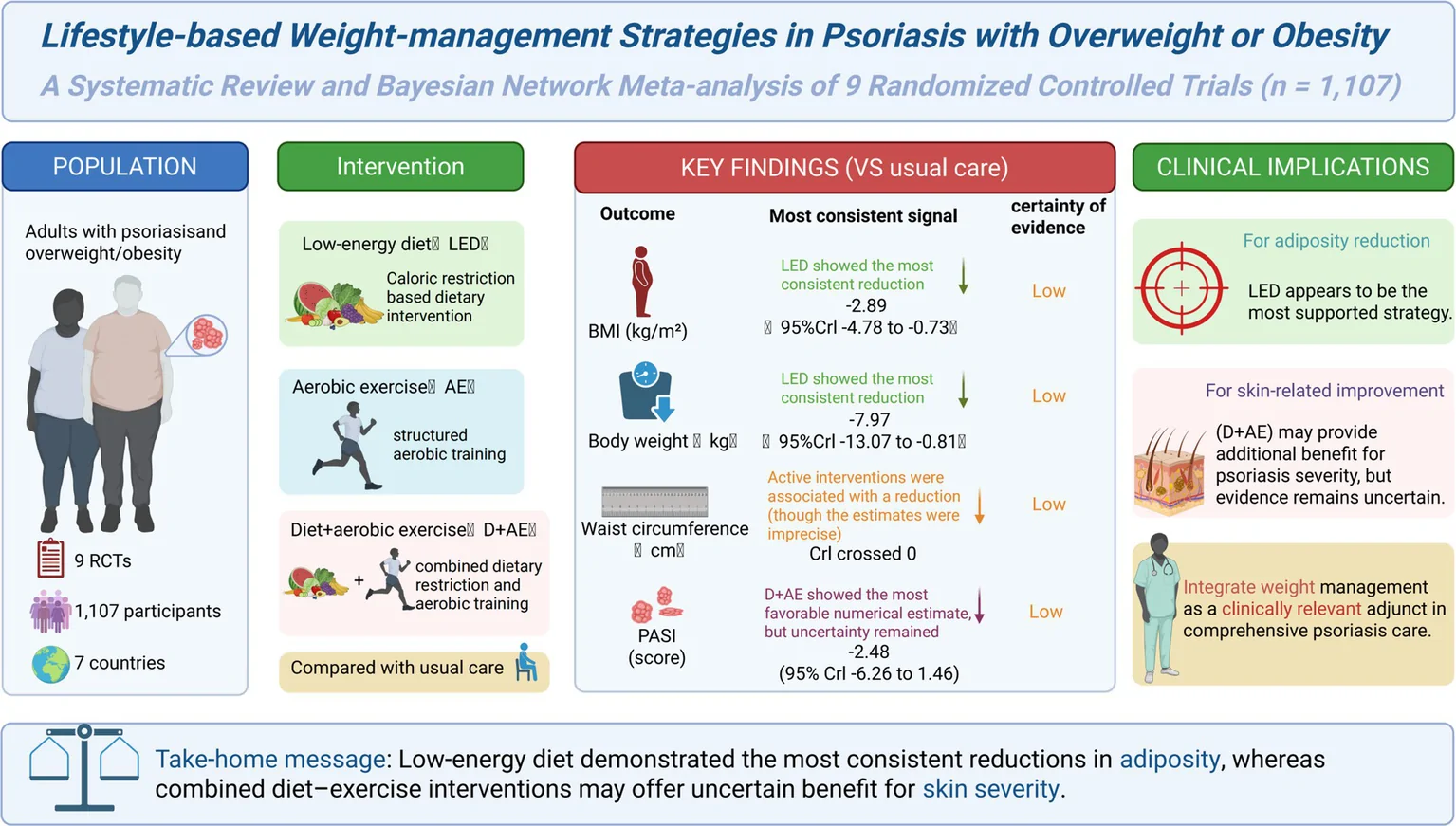

## Introduction

Excess adiposity is increasingly recognized as a modifiable driver of systemic inflammation and impaired treatment response in psoriasis and related obesity-associated inflammatory conditions (1–4). Psoriasis provides a compelling clinical example of this interaction. It is a chronic, immune-mediated inflammatory dermatosis affecting an estimated 60–125 million individuals worldwide (5, 6). Although traditionally regarded as a skin-limited disorder, psoriasis is now widely understood as a systemic inflammatory disease associated with a broad spectrum of cardiometabolic comorbidities, notably obesity and metabolic syndrome (1, 5, 7). Updated estimates from the Global Burden of Disease 2021 study indicate persistent increases in psoriasis burden over the past three decades, underscoring the need for management strategies that extend beyond skin-directed therapies (8). Among these comorbidities, adiposity is of particular clinical importance because it is common, clinically meaningful, and potentially modifiable.

The relationship between psoriasis and obesity appears to be bidirectional. Obesity has been associated with an increased risk of incident psoriasis, whereas psoriasis-associated psychosocial burden, reduced physical activity, and systemic inflammation may contribute to weight gain (1, 3, 5). Mechanistically, dysfunctional adipose tissue promotes chronic low-grade inflammation through adipokine imbalance, altered cytokine signaling, insulin resistance, and related metabolic dysregulation. These processes may in turn amplify psoriatic disease activity and impair therapeutic responsiveness (1–4). Observational and real-world evidence further suggests that elevated body mass index is associated with poorer treatment outcomes (4, 9, 10). Taken together, these findings position adiposity reduction not merely as a general health objective, but as a clinically relevant therapeutic target in psoriasis management (11, 12).

Dietary energy restriction remains a cornerstone of non-pharmacological obesity treatment. Previous syntheses have shown that low-calorie and very-low-energy dietary strategies can produce clinically meaningful short-term weight loss in populations with overweight or obesity (13–15). Beyond anthropometric changes, dietary interventions may also attenuate circulating inflammatory markers, including interleukin-6, suggesting potential immunometabolic benefits relevant to chronic inflammatory disease (16). Structured exercise may provide complementary effects through improvements in energy balance, insulin sensitivity, physical function, and cardiometabolic health (17, 18). Accordingly, combined diet–exercise programs are frequently advocated in individuals with obesity-related chronic conditions (11, 12).

In psoriasis, randomized trials and previous systematic reviews suggest that weight-loss and lifestyle interventions may improve body weight, disease severity, and health-related quality of life (19–23). However, most available syntheses have relied on pairwise comparisons of broad intervention categories against usual care or lower-intensity interventions. Such approaches provide limited insight into the relative effectiveness of specific lifestyle-based weight-management strategies and may obscure potentially different effects of dietary energy restriction, aerobic exercise, and combined interventions. This distinction is clinically important: the strategy that most effectively reduces adiposity may not necessarily yield the greatest improvement in psoriasis-specific outcomes. We therefore conducted a systematic review and Bayesian network meta-analysis to compare the relative effects of key weight-management strategies—dietary energy restriction, aerobic exercise, and combined diet–exercise interventions—on adiposity-related and psoriasis-specific outcomes in adults with psoriasis and overweight or obesity.

## Methods

### Reporting standards and protocol

The protocol for this systematic review and network meta-analysis (NMA) was registered in PROSPERO (CRD420261291403), and the review was conducted in accordance with the PRISMA 2020 statement and the PRISMA extension for network meta-analysis (PRISMA-NMA) (24, 25). Completed reporting checklists are provided in Appendix 1. The protocol was finalized before study screening and data extraction. To align the review more closely with its core objective of evaluating lifestyle-based weight-management strategies in psoriasis, adiposity-related indices were specified as primary outcomes before quantitative synthesis, while psoriasis-specific outcomes were retained as secondary outcomes. Other deviations from the registered protocol are summarized in Appendix 4.

### Eligibility criteria

Eligible studies were randomized controlled trials, including multi-arm trials, enrolling adults aged 18 years or older with a clinical or investigator-confirmed diagnosis of psoriasis or psoriatic arthritis who also had overweight or obesity, as defined by the original study investigators. Studies involving patients with psoriatic arthritis were eligible because psoriatic arthritis is part of the psoriatic disease spectrum and shares inflammatory and metabolic pathways with cutaneous psoriasis. Different clinical phenotypes were permitted, and no restrictions were imposed on disease severity at enrolment.

Eligible interventions were lifestyle-based weight-management strategies involving dietary energy restriction, structured exercise, or their combination. Comparators included usual care, routine clinical care, waitlist control, minimal lifestyle advice, attention or placebo conditions, or another eligible lifestyle intervention. Studies were required to report extractable data for at least one primary outcome: body mass index (BMI), body weight, or waist circumference. Secondary outcomes included the Psoriasis Area and Severity Index (PASI), the Dermatology Life Quality Index (DLQI), and safety outcomes.

Studies were excluded if the intervention duration was shorter than 1 week, if none of the predefined primary outcomes were reported, or if the available data were insufficient for effect estimation. No restrictions were imposed on language, publication status, or geographic location.

### Classification of interventions

A pre-specified treatment network was constructed by grouping interventions according to their primary lifestyle component into three active nodes: low-energy diet (LED), encompassing low-calorie diets and very-low-energy diets; diet combined with aerobic or walking exercise (D+AE); and aerobic exercise alone (AE). In the primary analysis, very-low-energy diet was not treated as a separate node but was included within the LED node. An exploratory sensitivity analysis examined the influence of this grouping.

Control interventions were grouped into a single usual care (UC) node, defined as routine clinical care, minimal lifestyle advice, waitlist control, or attention/placebo conditions, provided that they did not include explicit caloric restriction or a prescribed exercise program (26). Multi-arm trials were eligible for inclusion. Complex lifestyle interventions containing co-components, such as behavioral support or nutritional counseling, were included provided that the core component could be mapped to one of the pre-specified active nodes.

### Information sources and search strategy

The following English-language databases were searched from inception to 15 January 2026: PubMed, Ovid Embase, Web of Science, and Cochrane Library (CENTRAL). The following Chinese-language databases were additionally searched: CNKI, Wanfang, VIP, and CBM. No language restrictions were applied. Reference lists of included studies and relevant reviews were also hand-searched. Full search strategies for all databases are provided in Appendix 2.

### Study selection and data collection

Two reviewers (RH and WL) independently screened deduplicated titles and abstracts and assessed full-text reports for eligibility. Disagreements were resolved through discussion, with arbitration by a third reviewer (HL) when required. Data were independently extracted in duplicate by two reviewers (RH and NS) using a standardized form, and any unresolved disagreements were adjudicated by a third reviewer (TW). When key information was incomplete, trial registrations, protocols, and supplementary materials were consulted; original authors were contacted where necessary.

Publications by Jensen et al. (27, 28) reported different outcome sets from a 16-week randomized controlled trial conducted in a Danish population with psoriasis and obesity. The former primarily reported psoriasis-specific outcomes, whereas the latter reported anthropometric and cardiometabolic outcomes. Because each report contributed outcome data not fully represented in the other, both publications were retained in the quantitative synthesis to preserve the complete evidence base. The potential influence of this analytical choice was examined through pre-specified leave-one-out sensitivity analyses.

### Data extraction and intervention coding

Extracted information included sample size, follow-up duration, baseline disease severity, comorbidities, concomitant treatments, and intervention characteristics, including dietary energy restriction method and intensity, exercise modality, frequency, intensity, duration, and supervision status. The primary analysis time point was pre-defined as the end of the intervention period. When multiple follow-up time points were reported, the assessment closest to intervention completion was used.

### Risk of bias and certainty of evidence

Risk of bias was assessed using the Cochrane Risk of Bias 2 tool across five domains: the randomization process, deviations from intended interventions, missing outcome data, measurement of the outcome, and selection of the reported result (29). Certainty of evidence for network estimates was evaluated using the Confidence in Network Meta-Analysis (CINeMA) framework (30, 31).

### Statistical analysis

All analyses were conducted in R (version 4.5.1). Bayesian random-effects NMA was implemented using the multinma package (version 0.8.1), with usual care specified as the reference treatment (32). Continuous outcomes measured on a common scale were summarized as mean differences (MDs) with 95% credible intervals (CrIs), and between-study heterogeneity was quantified using the standard deviation of the random effects (*τ*) with its 95% CrI. Weakly informative priors were specified for treatment effects, intercepts, and heterogeneity. Sensitivity to the heterogeneity prior was assessed by varying the scale parameter of the half-normal distribution. Model convergence and fit were evaluated using R-hat statistics, effective sample sizes, and visual inspection of trace plots. Full prior specifications and Markov chain Monte Carlo settings are provided in Appendix 9. Clinical and methodological transitivity was assessed qualitatively by comparing study design, baseline participant characteristics, disease severity, intervention duration, concomitant psoriasis treatments, and usual care conditions across studies. Pre-specified network meta-regression analyses were then conducted using mean-centered intervention duration and baseline BMI as potential effect modifiers (32). Given the limited network size, these analyses were considered exploratory. Because the network was predominantly star-shaped with few closed loops, treatment rankings and indirect comparisons were interpreted cautiously. Global incoherence was explored by comparing the deviance information criterion of the consistency model with that of an unrelated mean effects model. Supplementary frequentist analyses were performed using the netmeta package (version 3.3.1) to assess the robustness of the Bayesian estimates and explore small-study effects. Comparison-adjusted funnel plots were examined, and formal asymmetry testing was planned only when at least 10 studies were available for a given comparison, in accordance with Cochrane Handbook recommendations (33).

### Sensitivity analyses

The following sensitivity analyses were pre-specified: exclusion of trials in which the control arm included explicit dietary adjustment or energy restriction; exclusion of trials in which background systemic therapy may have been imbalanced across arms; and an exploratory analysis examining the impact of grouping very-low-energy diet interventions within the LED node. Leave-one-out analyses were performed by sequentially excluding each study to assess the influence of individual trials on pooled estimates and heterogeneity. When exclusion of trials reduced the available evidence base to fewer than three studies per comparison, results are reported descriptively. For missing variance data, a pre-specified imputation hierarchy was applied; details are provided in Appendix 5 (33).

## Results

### Literature search

**Figure 1:**
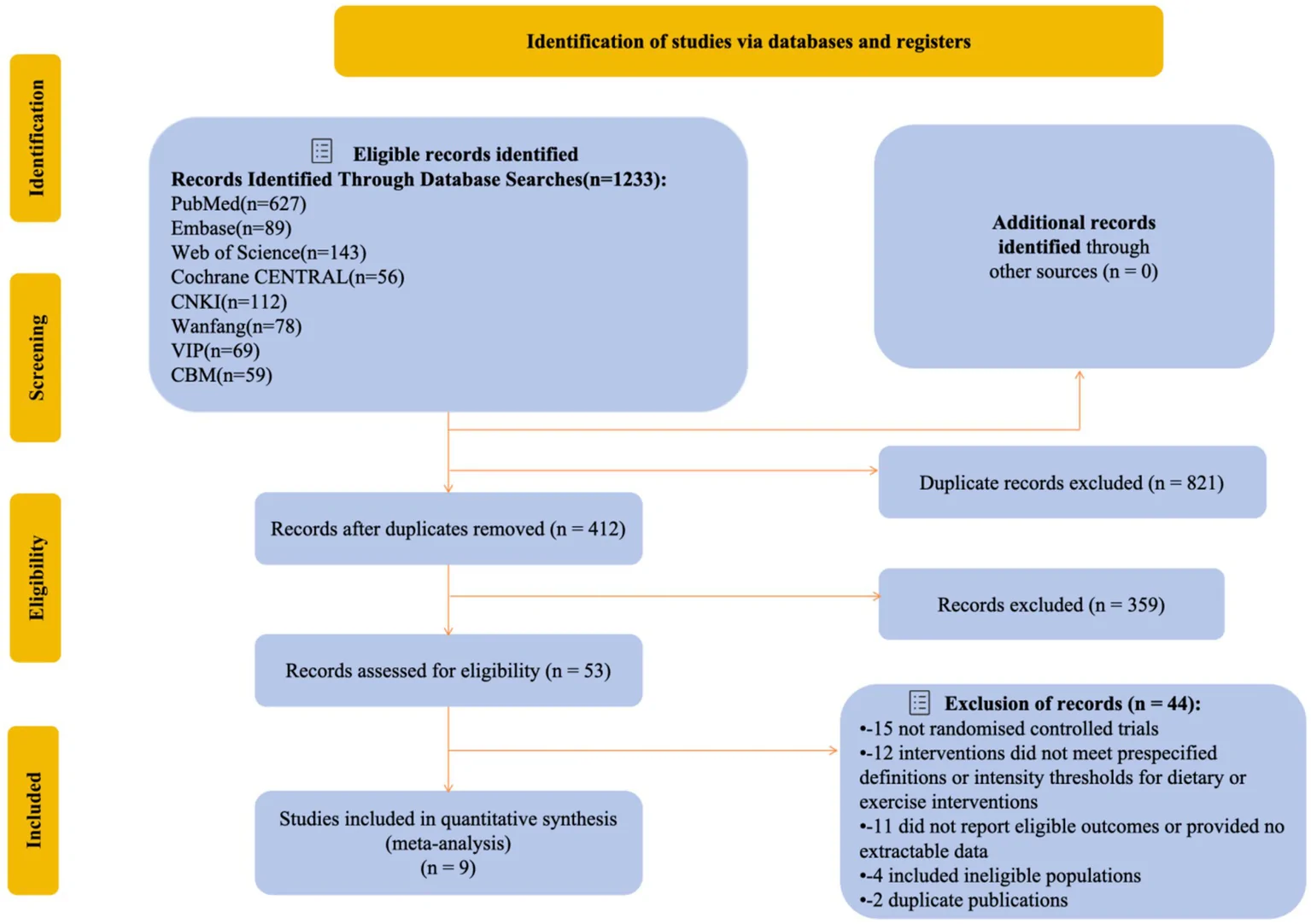
Flow chart of the search for eligible RCTs.

The database search identified 1,233 records. After removal of duplicates, 412 records underwent title and abstract screening, and 53 full-text articles were assessed for eligibility. Of these, 44 full-text reports were excluded: 15 were not randomized controlled trials, 12 evaluated interventions that did not meet the prespecified definitions or intensity thresholds for dietary or exercise interventions, 11 did not report eligible outcomes or did not provide extractable data, four included ineligible populations, and two were duplicate publications. Ultimately, nine RCTs were included in the quantitative synthesis (Figure 1).

### Study characteristics

The nine included RCTs enrolled 1,066 participants and were conducted across seven countries: Egypt, Denmark, Brazil, Italy, Spain, Sweden, and Kuwait (23, 27, 28, 34–39). Included populations comprised adults with cutaneous psoriasis, psoriatic arthritis, or psoriatic disease with overweight or obesity. Intervention duration ranged from 12 to 24 weeks, and baseline BMI across study arms ranged from 28.9 to 38.5 kg/m^2^, indicating that the available evidence was derived from populations with overweight or obesity (23, 27, 28, 34–39).

Based on the prespecified classification, four trials were assigned to low-energy diet (LED) (27, 36, 38, 39), four to diet plus aerobic or walking exercise (D+AE) (23, 28, 34, 35), and one to aerobic exercise alone (AE) (37). Usual care (UC) was the most common comparator, although its content varied across studies and included routine care, waitlist control, habitual diet, informative counseling, and minimal lifestyle advice (23, 27, 28, 34–39). The greatest clinical heterogeneity was observed within the D+AE node, which included different combinations of caloric restriction, walking, and supervised aerobic exercise (23, 28, 34, 35). In two trials, concomitant systemic therapy was administered in both study arms; this was explored in prespecified sensitivity analyses (35, 38). Detailed study characteristics are presented in Table 1.

**Table 1:** Characteristics of included studies.

Study	Country	Design	Intervention duration (weeks)	Number of participants (T/C)	Intervention	Dietary energy prescription	Comparator	Female participants (%)	Age, years (T/C)	BMI, kg/m^2^ (T/C)	Weight, kg (T/C)	Waist circumference (T/C)	Comorbidities	Blinding	Outcome measures
Ismail, 2024 (34)	Egypt	RCT	12	30/30	Low-calorie diet +treadmill walking	500 kcal/day energy deficit for 12 weeks	Waitlist	NA	44.03 ± 3.59/44.80 ± 3.17	31.85 ± 1.39/32.39 ± 1.38	NA	107.03 ± 14/109.46 ± 12.21	ED, MeTS, Obesity	Single-blinded	①③④
Ismail, 2023 (35)	Egypt	RCT	12	30/30	Low-calorie diet + walking	500 kcal/day energy deficit for 12 weeks	Immunosuppressive drugs only	NA	52 ± 11.20/51.33 ± 11.54	31.99 ± 3.92/32.60 ± 4.09	NA	109.78 ± 13.11/108.86 ± 13.13	NAFLD, Obesity	Open-label	①③④⑤
Landgren, 2025 (36)	Sweden	RCT	16	39/39	Very-low-energy diet (VLED) + exercise support	640 kcal/day for 12–16 weeks	Matched comparator group without psoriatic arthritis	64.1% / 74.4%	55 ± 10.37/56 ± 8.89	35.2 ± 2.97/38.5 ± 3.48	106.0 ± 14.07/107.0 ± 11.63	NA	Obesity	Open-label	①
Diaz, 2023 (37)	Spain	RCT	16	59/59	Aerobic training on treadmill	no dietary prescription	Control	NA	35.0 ± 6.1/37.8 ± 6.6	31.7 ± 3.9/32.2 ± 4.1	NA	NA	Psoriasis without PsA	Open-label	①
Leite, 2022 (38)	Brazil	RCT	12	32/33	Hypocaloric diet + placebo (DP) or omega 3 (DF)	500 kcal/day energy deficit for 12 weeks	Placebo + habitual diet	DP: 54.5%, DF: 54.8% / P: 54.5%	51.2 ± 15.1/53.6 ± 10.2	30.5 ± 6.2/28.9 ± 4.9	80.2 ± 16.1/76.2 ± 16.2	104.1 ± 14.3/101 ± 13.2	Diabetes, Hypertension, Dyslipidemia	Double-blinded	①②③④⑥
Naldi, 2014 (23)	Italy	RCT	20	151/152	Diet + physical exercise	80% of RMR for 12 weeks, then 100% of RMR for 8 weeks	Informative counseling	24.5% / 33.5%	53.0 ± 16.7/53.0 ± 21	30.8 ± 6.4/30.8 ± 6.0	90.0 ± 20.3/88.0 ± 20.7	112.0 ± 14.7/109.0 ± 16	Overweight, Obesity	Assessor-blind	①②③④⑥
Al-Mutairi, 2014 (39)	Kuwait	RCT	24	131/131	Low-calorie diet + biologics	≤1,000 kcal/day for 8 weeks	Normal diet + biologics	64.1% / 64.8%	44.5 ± 5.7/47.5 ± 6.9	29.3 ± 4.1/29.5 ± 5.1	99.3 ± 16.4/98.6 ± 17.9	102.5 ± 16.8/103.6 ± 18.3	Obesity	Open-label	①②③
Jensen, 2013 (27)	Denmark	RCT	16	30/30	Low-energy diet	800–1,000 kcal/day for 8 weeks, then 1,200 kcal/day for 8 weeks	Ordinary healthy foods	47% / 47%	50.7 ± 10.2/50.9 ± 10.6	34.7 ± 5.9/33.7 ± 4.5	106.7 ± 25.0/100.9 ± 19.1	111.3 ± 17.4/107.8 ± 10.2	Obesity	Unblinded	①②③
Jensen, 2014 (28)	Denmark	RCT	16	30/30	Low-energy diet	800–1,000 kcal/day for 8 weeks, then 1,200 kcal/day for 8 weeks	Ordinary healthy foods	47% / 47%	50.7 ± 10.2/50.9 ± 10.6	34.7 ± 5.9/33.7 ± 4.5	106.7 ± 25.0/100.9 ± 19.1	111.3 ± 17.4/107.8 ± 10.2	Diabetes, Hypercholesterolaemia, Hypertension	Unblinded	①②④

T/C, treatment group/control group; w, weeks; NA, not available; ED, erectile dysfunction; MeTS, metabolic syndrome; NAFLD, non-alcoholic fatty liver disease; RMR, resting metabolic rate; LCD, low-calorie diet; LED, low-energy diet; VLED, very-low-energy diet; PsA, psoriatic arthritis; NR, not reported. ① BMI, Body Mass Index; ② Weight, Body Weight; ③ WC, Waist Circumference; ④ PASI, Psoriasis Area and Severity Index; ⑤ DLQI, Dermatology Life Quality Index; ⑥ Safety outcomes, adverse events, serious adverse events, and withdrawal.

### Risk of bias

Among the nine included RCTs, three were judged to be at overall low risk of bias (36–38), whereas the remaining six were rated as having some concerns. No study was judged to be at high risk of bias. All studies were assessed as low risk for missing outcome data and measurement of the outcome.

**Figure 2:**
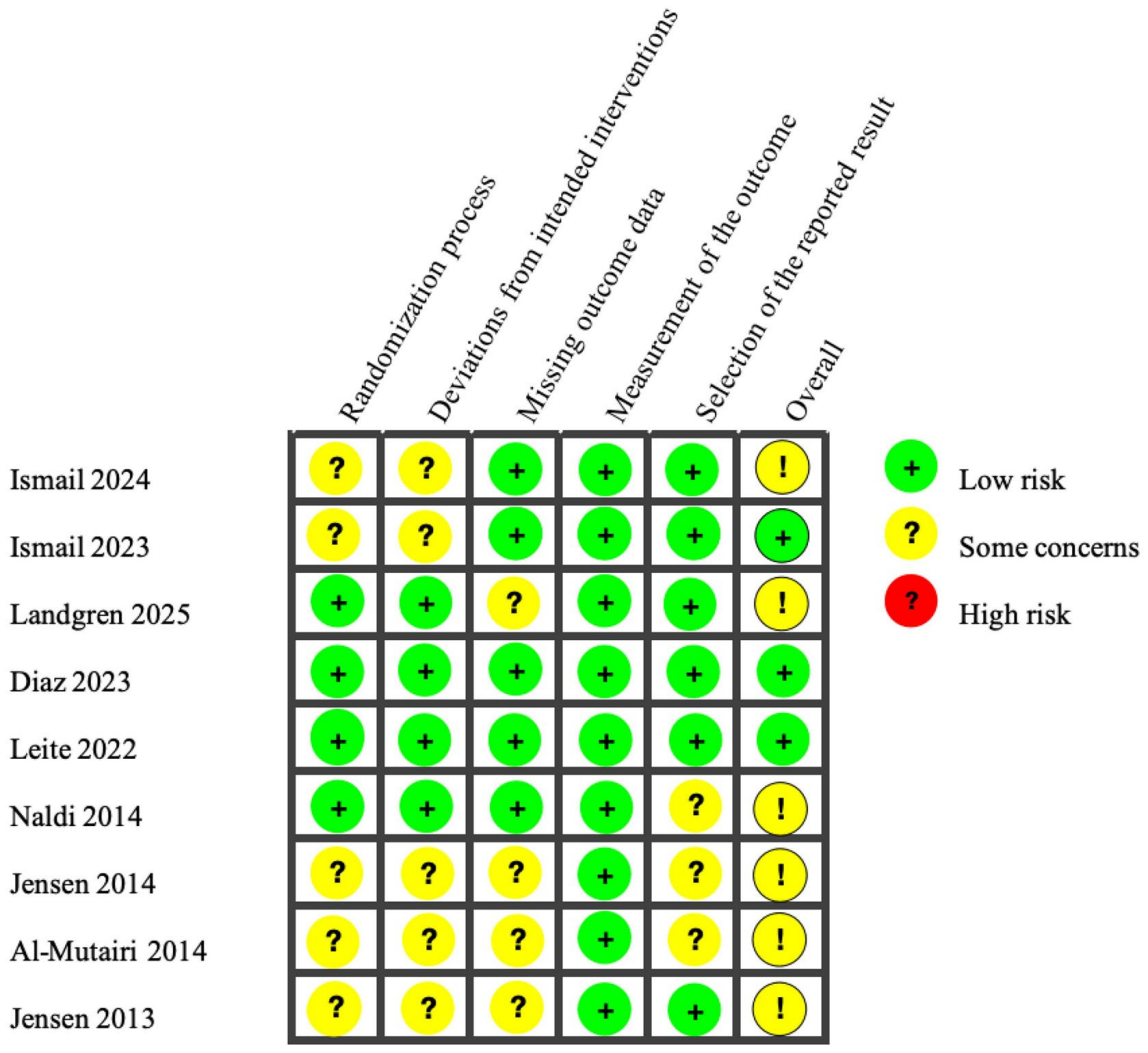
Assessment of risk of bias.

The main sources of concern arose from the randomization process and deviations from intended interventions, with six studies rated as having some concerns in both domains (23, 27, 28, 34, 35, 39). These judgments were primarily driven by limited reporting of sequence generation, allocation concealment, and the handling of departures from assigned interventions in the analysis. Concerns regarding selection of the reported result were identified in three studies (23, 28, 39), reflecting insufficient information on prespecified analysis plans or trial registration. Overall, no included study was judged to be at high risk of bias, but several studies had reporting limitations related to trial conduct and prespecified analysis plans. Detailed domain-level assessments are shown in Figure 2.

### Network structure

**Figure 3:**
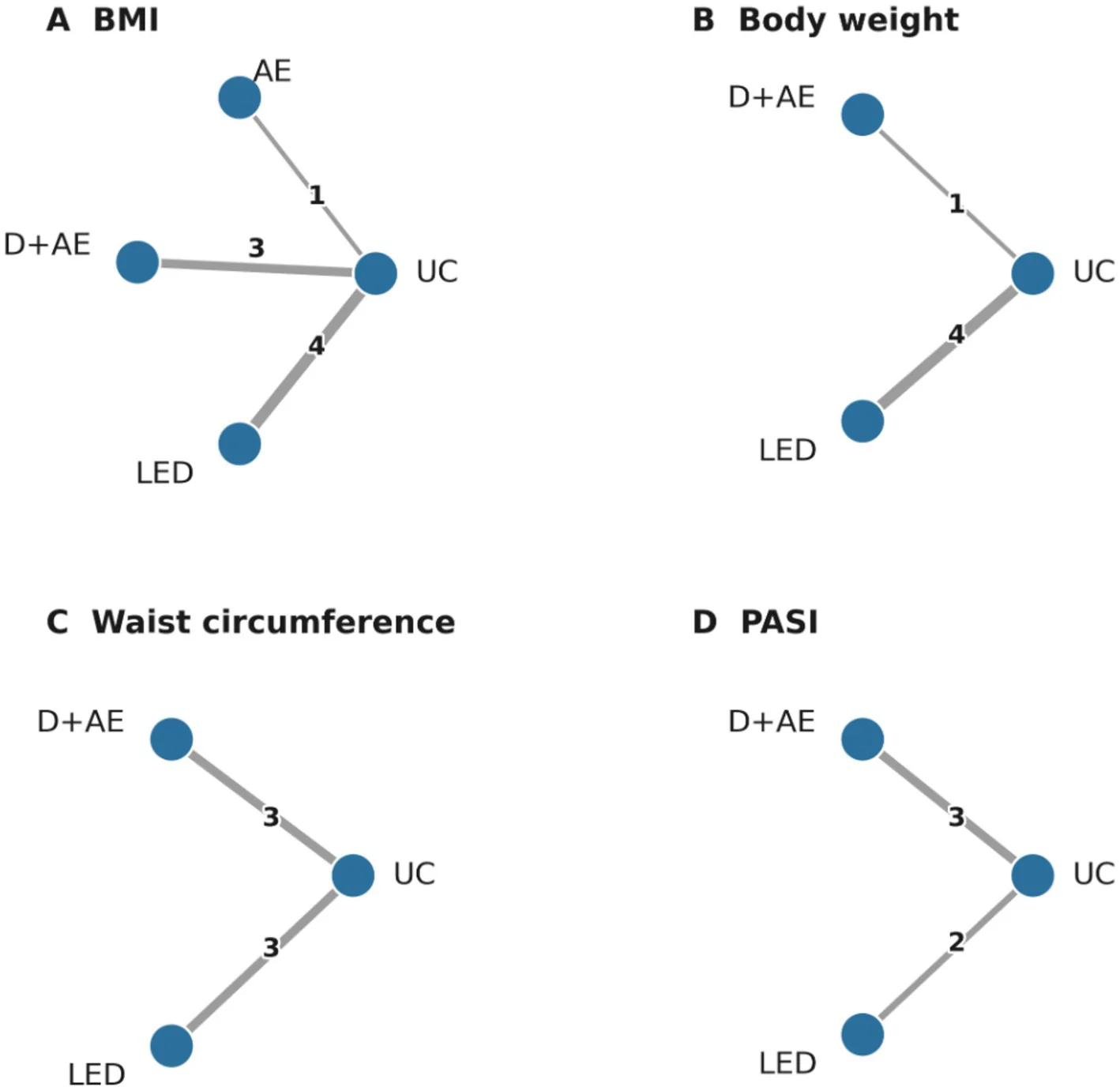
Network plots for primary and secondary outcomes. Network plots for **(A)** BMI, **(B)** body weight, **(C)** waist circumference, and **(D)** PASI. Nodes represent interventions, with node size proportional to the number of randomized participants, and lines represent direct comparisons. The marked differences in node size reflect considerable disparities in sample size across intervention nodes, particularly the substantially smaller AE node. Line thickness is proportional to the number of trials contributing to each comparison, and the numbers on the lines indicate the corresponding study counts. AE, aerobic exercise alone; D+AE, diet combined with aerobic or walking exercise; LED, low-energy diet; UC, usual care.

The network geometry was sparse and predominantly star-shaped across the analyzed outcomes, with usual care (UC) serving as the central comparator in all networks (Figure 3). For BMI, direct evidence connected UC with AE, D+AE, and LED, with one trial contributing to the AE node, three to the D+AE node, and four to the LED node. For body weight, the network consisted of D+AE versus UC and LED versus UC, informed by one and four trials, respectively. For waist circumference, direct evidence was available for D+AE versus UC and LED versus UC, with three trials informing each comparison. The PASI network included D+AE, LED, and UC, with no direct head-to-head comparisons between active interventions. Given the absence of closed loops and the limited direct evidence between active strategies, indirect comparisons and treatment rankings were interpreted cautiously.

### Primary outcomes

#### BMI

**Figure 4:**
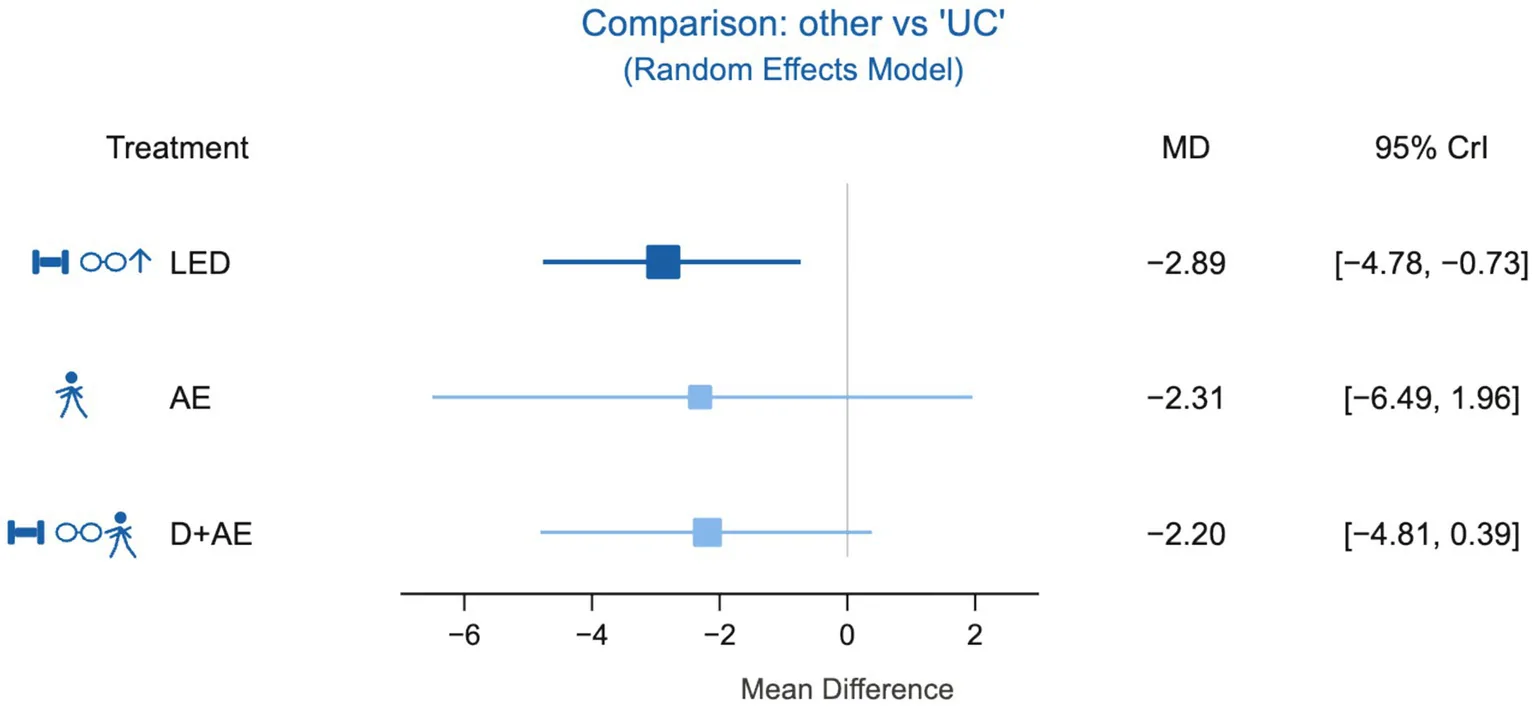
Effects of lifestyle interventions on BMI compared with usual care.

In the primary Bayesian random-effects NMA, all active interventions showed point estimates favoring BMI reduction versus UC, although the precision of estimates varied across comparisons. LED showed the most consistent evidence of benefit (MD, −2.89 kg/m^2^; 95% CrI, −4.78 to −0.73; Figure 4; Appendix Table S10.1). D+AE showed a numerically favorable effect on BMI reduction, whereas the estimate for AE was highly uncertain, with a wide credible interval including the null. Notably, the AE estimate was informed by only a single randomized controlled trial and should therefore be interpreted as exploratory. Indirect comparisons among active interventions showed no credible differences (Appendix Table S11.1). Supplementary frequentist random-effects analyses were broadly consistent with the Bayesian findings. LED remained superior to UC (MD, −3.41 kg/m^2^; 95% CI, −4.76 to −2.05), and D+AE also reached statistical significance (MD, −2.19 kg/m^2^; 95% CI, −3.64 to −0.74), whereas the estimate for AE was not statistically significant (Appendix Table S14.2). Between-study heterogeneity was moderate to high (*τ* = 1.87; 95% CrI, 0.49 to 4.27; Appendix Table S9.2). According to CINeMA, confidence in the evidence for BMI comparisons was low, mainly because of imprecision and heterogeneity (Appendix Table S12.2).

#### Body weight

**Figure 5:**
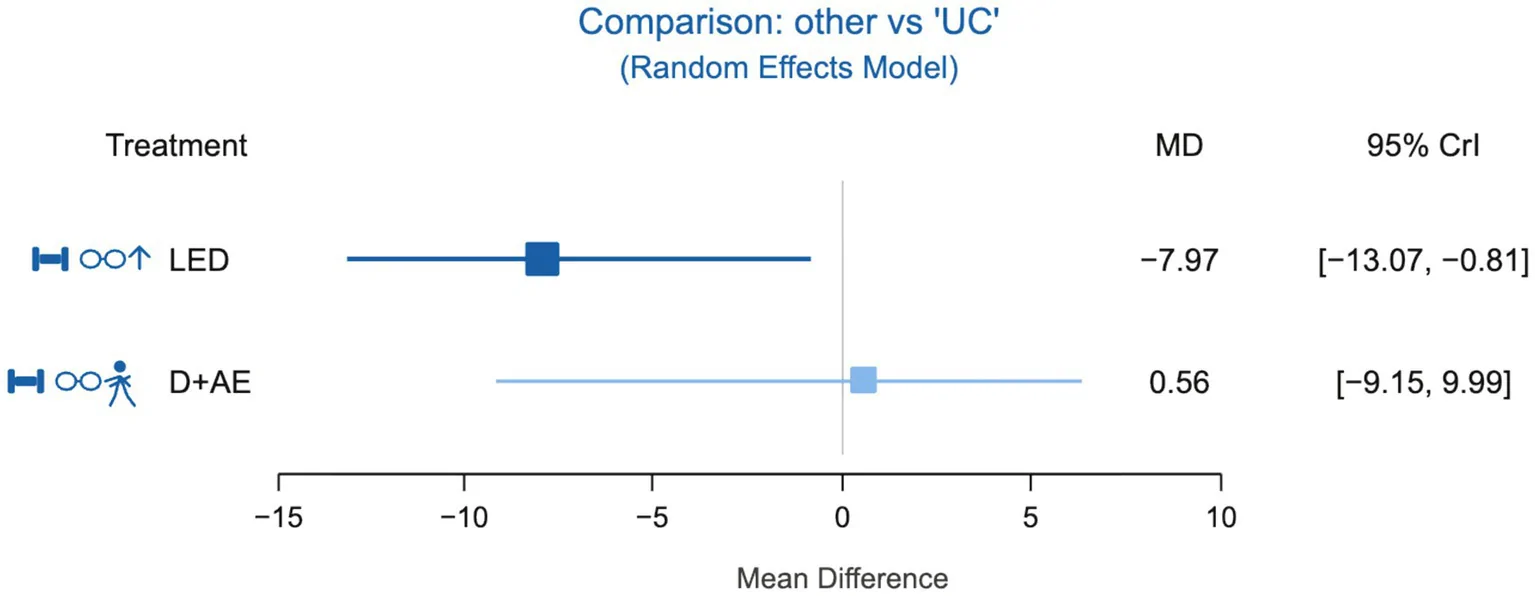
Effects of lifestyle interventions on body weight compared with usual care.

In the primary Bayesian random-effects NMA, LED showed the most consistent evidence of body-weight reduction versus UC (MD, −7.97 kg; 95% CrI, −13.07 to −0.81; Figure 5; Appendix Table S10.2). The estimate for D+AE versus UC was centered close to the null and was highly imprecise (MD, 0.56 kg; 95% CrI, −9.15 to 9.99). The indirect comparison between LED and D+AE showed no credible difference (Appendix Table S11.2).

Supplementary frequentist random-effects analyses were broadly consistent with the Bayesian findings. LED remained superior to UC (MD, −8.38 kg; 95% CI, −14.01 to −2.75), whereas the estimate for D+AE was not statistically significant (Appendix Table S14.2). Between-study heterogeneity was high (*τ* = 4.97; 95% CrI, 0.87 to 10.20; Appendix Table S9.2). According to CINeMA, confidence in the evidence for body-weight comparisons was low, mainly because of imprecision and heterogeneity (Appendix Table S12.3).

#### Waist circumference

**Figure 6:**
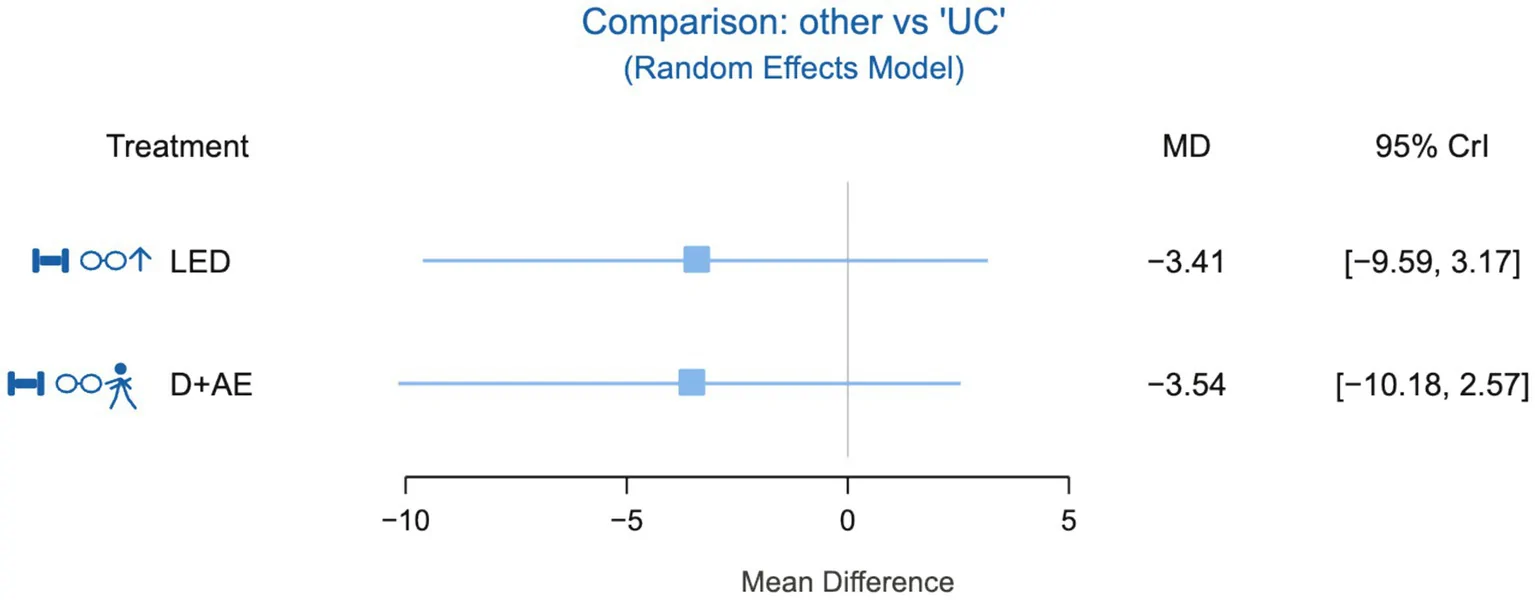
Effects of lifestyle interventions on waist circumference compared with usual care.

In the primary Bayesian random-effects NMA, both active interventions yielded point estimates favoring waist-circumference reduction compared with UC, although uncertainty was substantial. D+AE showed an MD of −3.54 cm (95% CrI, −10.18 to 2.57; Figure 6; Appendix Table S10.3), whereas LED showed an MD of −3.41 cm (95% CrI, −9.59 to 3.17). Because the 95% CrIs for both comparisons included the null, neither intervention showed credible evidence of superiority over UC. The indirect comparison between D+AE and LED also showed no credible difference (Appendix Table S11.3).

Supplementary frequentist random-effects analyses were broadly consistent with the Bayesian findings. Both active interventions yielded point estimates favoring waist-circumference reduction, but neither comparison was statistically significant (Appendix Table S14.2). Between-study heterogeneity was high (*τ* = 5.08; 95% CrI, 2.21 to 9.58; Appendix Table S9.2). According to CINeMA, confidence in the evidence for waist-circumference comparisons was low, mainly because of imprecision and heterogeneity (Appendix Table S12.4).

### Secondary outcomes

#### PASI

**Figure 7:**
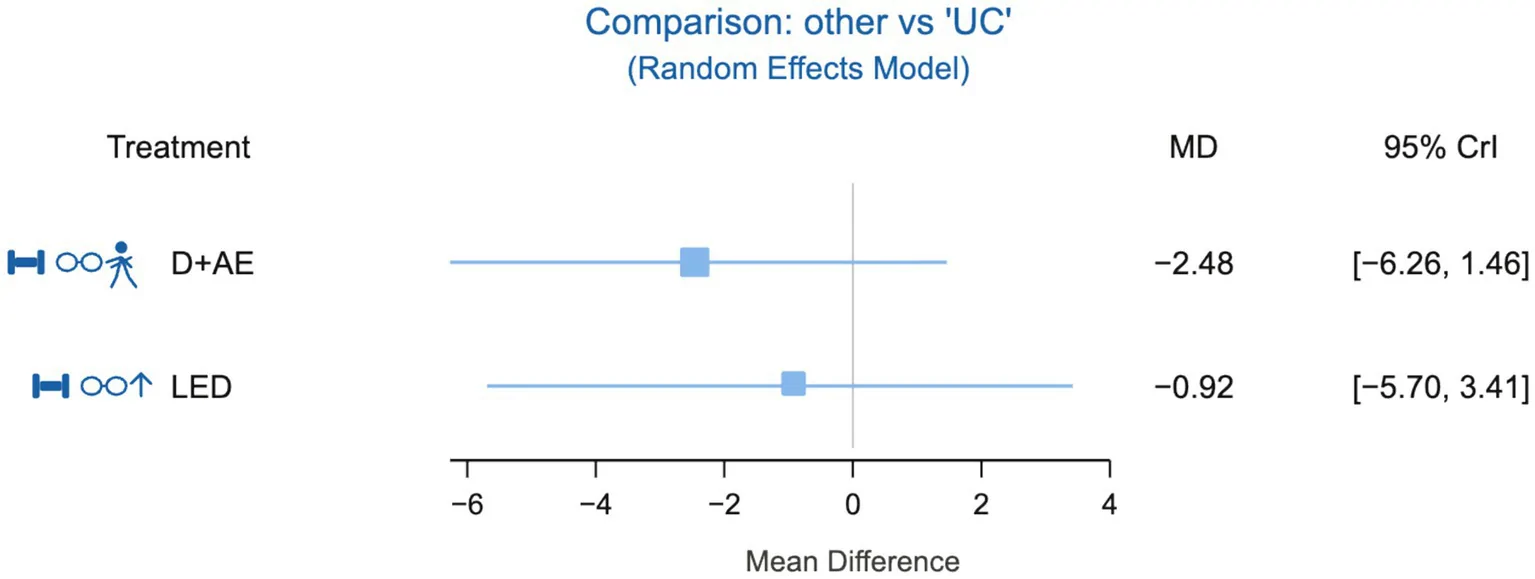
Effects of lifestyle interventions on PASI compared with usual care.

In the primary Bayesian random-effects NMA, both active interventions yielded point estimates favoring PASI reduction compared with UC, although substantial uncertainty remained. D+AE showed the more favorable point estimate versus UC (MD, −2.48; 95% CrI, −6.26 to 1.46; Figure 7; Appendix Table S10.4), whereas LED showed a smaller and more imprecise effect estimate (MD, −0.92; 95% CrI, −5.70 to 3.41). Because the 95% CrIs for both comparisons included the null, neither lifestyle strategy showed credible evidence of superiority over UC for improving PASI. The indirect comparison between D+AE and LED also showed no credible difference (Appendix Table S11.4).

Supplementary frequentist random-effects analyses were broadly consistent with the Bayesian findings. D+AE showed a point estimate favoring PASI reduction versus UC, whereas the estimate for LED was closer to the null; however, neither comparison was statistically significant, and the available evidence remained insufficient to determine whether either lifestyle intervention improved psoriasis severity (Appendix Table S14.2). Between-study heterogeneity was moderate to high (*τ* = 2.44; 95% CrI, 0.96 to 6.65; Appendix Table S9.2). According to CINeMA, confidence in the evidence for PASI comparisons was low, mainly because of imprecision and heterogeneity (Appendix Table S12.5).

### DLQI and safety outcomes

DLQI and safety data were too sparse and inconsistently reported to permit quantitative synthesis and were therefore summarized narratively (Appendix Table S17.1). DLQI was reported in only one trial, in which the intervention group showed a between-group improvement exceeding the commonly cited minimal clinically important difference of 4 points compared with usual care. This finding should be interpreted cautiously because it was based on a single study and was insufficient to support firm conclusions regarding dermatology-specific quality of life.

Safety reporting was limited and heterogeneous across trials. Where reported, adverse events were generally mild and self-limiting, most commonly hunger, fatigue, and constipation, and occurred more frequently in intervention groups. No serious intervention-related adverse events, hospitalizations, or deaths were reported in the included studies.

### Sensitivity analyses

Sensitivity analyses were broadly consistent with the primary findings. For BMI, exclusion of trials with potentially imbalanced background systemic therapy did not materially change the overall pattern of treatment effects. In leave-one-out analyses, the DIETA trial (38) was the main contributor to between-study heterogeneity for both BMI and body weight, as its exclusion produced the greatest reduction in *τ*. For waist circumference, the largest reduction in heterogeneity was observed after exclusion of Naldi et al. (23). Although these studies influenced heterogeneity estimates, their exclusion did not materially alter the direction or overall interpretation of the primary treatment effects (Appendix Figures S16.1–S16.3; Appendix Tables S16.1–S16.3).

## Discussion

### Principal findings

This systematic review and Bayesian network meta-analysis compared lifestyle-based weight-management strategies in adults with psoriasis and overweight or obesity across four outcomes: BMI, body weight, waist circumference, and PASI. The most consistent evidence was observed for low-energy diet, which ranked highest for BMI and body weight reduction relative to usual care, although the certainty of evidence was low. For waist circumference, point estimates also favored active interventions, but credible intervals were wide and uncertainty remained substantial. Diet plus aerobic exercise yielded the most favorable numerical estimate for PASI reduction, although the corresponding credible interval crossed the null and no strategy demonstrated a statistically credible advantage over usual care for this outcome. Taken together, these findings suggest that lifestyle-based weight-management strategies may have partially distinct effects on adiposity-related and psoriasis-specific outcomes, and that no single strategy emerged as clearly superior across all endpoints.

### Interpretation of findings

The apparent advantage of low-energy diet for BMI and body weight is biologically and clinically plausible. In obesity, dysfunctional adipose tissue functions as an active inflammatory organ. It increases the secretion of pro-inflammatory adipokines and cytokines while impairing anti-inflammatory signaling. Leptin may promote dendritic cell activation and Th17 differentiation, thereby linking excess adiposity to psoriatic inflammation (40, 41). Obesity may consequently amplify chronic low-grade inflammation and worsen psoriasis activity (40–42). Dietary energy restriction may attenuate these processes by reducing adiposity, which may partly explain the more consistent anthropometric effects observed with low-energy diets. However, the present study was not designed to test biological mechanisms. These interpretations should therefore be regarded as mechanistic context rather than direct causal evidence.

A notable finding was that diet plus aerobic exercise yielded a more favorable numerical estimate for PASI than low-energy diet, despite the clearer effects of the latter on BMI and body weight. This pattern raises the possibility that structured exercise may act through mechanisms not fully captured by weight reduction alone. Exercise may promote myokine release and improve insulin sensitivity and cardiorespiratory function. It may also reduce systemic oxidative stress and inflammation (18, 43, 44). Nevertheless, PASI estimates were imprecise, and the credible intervals were compatible with no clear between-strategy difference. This finding should therefore be interpreted cautiously and regarded as hypothesis-generating rather than confirmatory.

For waist circumference, both active interventions produced directionally favorable point estimates relative to usual care, but neither estimate was statistically credible. Waist circumference is a clinically relevant marker of central adiposity, yet it is more susceptible than body weight to measurement variability arising from differences in anatomical landmarking, posture, respiratory phase, and assessor technique (45). Such variability may have contributed to the observed imprecision. In addition, the aerobic exercise alone node was informed by only one trial, resulting in considerable statistical instability. Any inference regarding aerobic exercise in isolation should therefore remain cautious and exploratory.

### Heterogeneity

Between-study heterogeneity was moderate to high across outcomes. Sensitivity analyses identified the DIETA trial by Leite et al. as an important contributor to heterogeneity for BMI and body weight (38). This is plausible because the intervention in that trial differed from other low-energy diet studies in intensity, co-intervention structure, and implementation context. Very-low-energy diet protocols are commonly defined as providing 800 kcal per day or fewer and may produce larger short-term weight-loss effects than conventional calorie-restriction approaches (13, 46). For waist circumference, excluding Naldi et al. produced the largest reduction in heterogeneity (23). This suggests that differences in intervention intensity, behavioral support, comparator content, or outcome assessment may have contributed to the observed inconsistency. Importantly, although individual studies influenced heterogeneity estimates, their exclusion did not materially alter the direction or overall interpretation of the primary treatment effects.

### Clinical implications

These findings have several implications for clinical practice. In adults with psoriasis and overweight or obesity, low-energy diet showed the most consistent evidence for reducing BMI and body weight and may therefore be particularly relevant when short-term adiposity reduction or improvement in obesity-related metabolic risk is the primary therapeutic objective. Given the well-recognized association between obesity, psoriasis severity, and reduced treatment responsiveness, the present findings support weight management as a clinically relevant adjunct in comprehensive psoriasis care (40, 47, 48).

At the same time, the more favorable numerical estimate for PASI observed with diet plus aerobic exercise suggests that combined approaches may warrant further investigation for potential effects on skin-related outcomes. However, this finding remains uncertain, and current evidence is insufficient to determine whether lifestyle interventions independently improve psoriasis severity. Therefore, lifestyle strategies should be considered as adjunctive approaches that complement established psoriasis treatments, rather than replacements for pharmacological disease-directed therapy. This highlights the need to individualize lifestyle prescriptions according to the primary treatment goal, patient preference, feasibility, and disease-related barriers.

Exercise prescription in psoriasis may require additional adaptation because plaque discomfort, joint symptoms, fatigue, weight stigma, and psychosocial concerns can reduce adherence to standard programs (49–51). Flexible, patient-centered, and barrier-adapted models may therefore be important for improving long-term engagement and implementation (50, 51).

### Sustainability and maintenance of intervention effects

The sustainability of the observed effects remains uncertain because the included studies primarily assessed outcomes over intervention periods of 12 to 24 weeks, with limited follow-up after active treatment ended. Accordingly, it remains unclear whether the observed reductions in BMI and body weight, or any potential changes in psoriasis severity, are maintained once structured dietary support or exercise supervision is withdrawn. This issue is particularly relevant in psoriasis, which requires long-term disease management and sustained control of obesity-related risk. Nevertheless, the short-term anthropometric benefits identified in this review remain clinically relevant and provide an important basis for developing longer-term weight-management strategies. Future trials should evaluate not only initial treatment effects but also their maintenance after the active intervention period, including sustained adherence, weight regain, and persistence of psoriasis-related outcomes.

### Strengths and limitations

To our knowledge, this is the first network meta-analysis to compare clinically distinct lifestyle-based weight-management strategies in psoriasis while evaluating both adiposity-related and psoriasis-specific outcomes. By moving beyond broad intervention-versus-control comparisons, this approach provides comparative estimates that may help inform future trial design, although the findings should be interpreted in light of the limited direct evidence.

Several limitations should be acknowledged. First, the certainty of evidence was low across outcomes because of imprecision, between-study heterogeneity, and the sparse, star-shaped network. The absence of closed loops and direct head-to-head comparisons meant that comparisons between active interventions relied entirely on indirect evidence, limiting inconsistency assessment and the precision of comparative estimates (52, 53). Accordingly, indirect comparisons between active interventions should be regarded as hypothesis-generating rather than confirmatory evidence for clinical decision-making. Second, variation in comparator conditions and intervention intensity introduced additional uncertainty regarding the transitivity assumption. Comparator groups ranged from waitlist and pharmacotherapy-only controls to informational counseling and attention-matched dietary guidance, while the low-energy diet node combined very-low-energy diets (≤800 kcal/day), less restrictive low-calorie diets, and individualized energy-deficit prescriptions. These interventions differed in intensity, expected rate of weight loss, supervision requirements, and safety considerations, which may have influenced treatment contrasts and contributed to heterogeneity. Because few studies were available within each caloric-restriction category, reliable stratified analyses were not feasible. Accordingly, the findings should not be interpreted as supporting a uniform prescription of low-energy diets; clinical application should consider the intensity and duration of energy restriction, patient characteristics, professional supervision, and potential safety concerns. Detailed comparator characteristics are provided in Appendix Table S8.3. Third, the aerobic exercise node was informed by only one trial and should therefore be regarded as exploratory. Fourth, network meta-regression was underpowered to reliably identify effect modification. Fifth, Jensen et al. (27, 28) reported different outcome sets from the same underlying trial and were retained without double-counting the same outcome data; leave-one-out sensitivity analyses did not materially alter the pooled estimates or direction of effects. Sixth, the short intervention periods and limited post-intervention follow-up prevented assessment of whether the observed anthropometric effects were maintained or translated into sustained improvements in psoriasis-related outcomes. Finally, DLQI and safety data were sparse and inconsistently reported, and adverse events were not systematically defined or monitored across trials. Therefore, this review cannot provide a reliable safety assessment of intensive dietary restriction or exercise regimens, and individualized clinical monitoring is warranted in practice, particularly when substantial caloric restriction is prescribed or relevant comorbidities are present.

### Future research

Future trials should directly compare low-energy diet, structured aerobic exercise, and combined diet–exercise interventions using standardized intervention definitions and harmonized outcome sets (49, 54). In addition to PASI and anthropometric measures, consistent reporting of DLQI, physical activity, adverse events, adherence, and concomitant psoriasis treatments would improve the clinical interpretability and applicability of the evidence base (49, 55). Future studies should include at least 6 to 12 months of post-intervention follow-up, ideally with repeated assessments after structured intervention support has ended. Such follow-up would allow investigators to evaluate the durability of lifestyle changes, the extent of weight regain, sustained adherence, persistence of any improvement in psoriasis severity, and the longer-term risk of psoriasis relapse or treatment escalation (56).

Glucagon-like peptide-1 receptor agonists (GLP-1 RAs) and related incretin-based therapies have emerged as highly effective pharmacological options for weight management. Their use introduces a further dimension to adiposity-targeted care in chronic inflammatory diseases. Preliminary evidence suggests that GLP-1 RAs may exert anti-inflammatory effects beyond weight reduction alone. Early clinical observations have also raised the possibility of benefits in psoriatic disease (57, 58). Given that dietary energy restriction produced the most consistent adiposity reductions in the present review, future studies should examine how lifestyle and pharmacological weight-management strategies can be integrated. Trials could compare lifestyle intervention alone, pharmacological weight management alone, and combined approaches. Such studies would help determine whether these strategies have additive or synergistic effects on metabolic and dermatological outcomes in people with psoriasis and overweight or obesity.

## Conclusion

Lifestyle-based weight-management strategies generally favored improvements over usual care in adults with psoriasis and overweight or obesity. Low-energy diet showed the most consistent evidence for reducing BMI and body weight, whereas combined diet–exercise interventions yielded the most favorable numerical estimate for PASI, although this finding remained uncertain. Overall, these findings support weight management as a clinically meaningful adjunct in comprehensive psoriasis care and highlight the need for adequately powered head-to-head trials to identify the most appropriate lifestyle strategy for different clinical priorities.

## Data Availability

The original contributions presented in the study are included in the article/Supplementary material, further inquiries can be directed to the corresponding author.
