# Supplementary material for: Lifestyle interventions targeting adiposity in adults with psoriasis: a systematic review and network meta-analysis
Source: Front Nutr. 2026 Aug 6;13:1912400. doi: 10.3389/fnut.2026.1912400 (PMC13491646; doi:10.3389/fnut.2026.1912400)
Supplement: Supplementary file 1 [file Supplementary_file_1.docx]

**Supplementary Appendix**

**Lifestyle interventions targeting adiposity in adults with psoriasis: a systematic review and network meta-analysis**

**Table of contents**

**[Appendix 1:](#_Toc1346393294)** [Checklist for Reporting Network Meta-Analyses (PRISMA-NMA Extension)	2](#_Toc1346393294)

**[Appendix 2:](#_Toc796903819)** [Search strategy](#_Toc796903819)	7

**[Appendix 3:](#_Toc1854463241)** [Pre-specified classification framework for intervention nodes included in the network meta-analysis	1](#_Toc1854463241)2

**[Appendix 4:](#_Toc1533522576)** [Deviations from the registered protocol	1](#_Toc1533522576)4

**[Appendix 5:](#_Toc1962687185)** [Pre-specified imputation hierarchy for missing variance data](#_Toc1962687185)

[1](#_Toc1962687185)6

**[Appendix 6:](#_Toc1534700375)** [Full text articles excluded after full text review with reasons for exclusion](#_Toc1534700375)

[1](#_Toc1534700375)7

**[Appendix 7:](#_Toc283118508)** [Risk of bias of randomized clinical trials](#_Toc283118508)

[2](#_Toc283118508)4

**[Appendix 8:](#_Toc1696485851)** [Data extraction framework, variable definitions, and data handling rules for the network meta-analysis.	2](#_Toc1696485851)7

**[Appendix 9:](#_Toc697316538)** [Bayesian prior specification, MCMC settings, convergence diagnostics, heterogeneity, and model fit.	3](#_Toc697316538)2

**[Appendix 10:](#_Toc980792487)** [Bayesian network meta-analysis results, SUCRA, and cumulative ranking probability plots](#_Toc980792487)

[.	3](#_Toc980792487)4

**[Appendix 11:](#_Toc94854637)** [League tables of Bayesian network meta-analysis results for lifestyle interventions in psoriasis	3](#_Toc94854637)8

**[Appendix 12:](#_Toc789017985)** [CINeMA Assessment	3](#_Toc789017985)9

**[Appendix 13:](#_Toc313753670)** [Funnel plots
.	4](#_Toc313753670)7

**[Appendix 14:](#_Toc2134232375)** [Frequentist sensitivity analysis](#_Toc2134232375)

[.](#_Toc2134232375)	50

**[Appendix 15:](#_Toc624170784)** [Meta-regression analyses](#_Toc624170784)

[5](#_Toc624170784)2

**[Appendix 16:](#_Toc2128234740)** [Leave-one-out sensitivity analyses.	5](#_Toc2128234740)4

**[Appendix 17:](#_Toc2128234740)** [Narrative summary of DLQI and safety outcomes](#_Toc2128234740)

[58](#_Toc2128234740)

**Appendix 1: Checklist for Reporting Network Meta-Analyses (PRISMA-NMA Extension)**

| **Section/Topic** | **Item #** | **Checklist Item** | **Reported on Page #** |
| --- | --- | --- | --- |
| **TITLE** |  |  |  |
| Title | 1 | Identify the report as a systematic review incorporating a network meta-analysis. | ***1*** |
|  |  |  |  |
| **ABSTRACT** |  |  | ***1*** |
| Structured summary | 2 | Provide a structured summary including, as applicable:  **Background:** main objectives;  **Methods:** data sources, study eligibility criteria, participants, interventions, risk-of-bias assessment, and synthesis methods, including Bayesian network meta-analysis;  **Results:** number of included studies and participants, summary estimates with corresponding credible intervals, and treatment rankings if reported; **Discussion/Conclusions:** limitations, interpretation of findings, and clinical implications;  **Other:** funding source and systematic review registration number. |  |
|  |  |  |  |
| **INTRODUCTION** |  |  |  |
| Rationale | 3 | Describe the rationale for the review in the context of what is already known*, including mention of why a network meta-analysis has been conducted.* | ***2-3*** |
| Objectives | 4 | Provide an explicit statement of questions being addressed, with reference to participants, interventions, comparisons, outcomes, and study design (PICOS). | 3 |
|  |  |  |  |
| **METHODS** |  |  |  |
| Protocol and registration | 5 | Indicate whether a review protocol exists and if and where it can be accessed (e.g., Web address); and, if available, provide registration information, including registration number. | ***3-4*** |
| Eligibility criteria | 6 | Specify study characteristics (e.g., participants, interventions, comparators, outcomes, follow-up) and report characteristics (e.g., years considered, language, publication status) used as criteria for eligibility, with rationale. *Clearly describe the interventions included in the treatment network, including any clustering of related interventions into common nodes, with justification.* | 4-5 |
| Information sources | 7 | Describe all information sources (e.g., bibliographic databases, reference lists, trial registrations, contact with study authors) and the date last searched. | 5 |
| Search | 8 | Present the full electronic search strategy for at least one database, including any limits used, such that it can be reproduced. | 5; Appendix 2 |
| Study selection | 9 | State the process for study selection (i.e., screening, assessment of eligibility, and inclusion in the systematic review and network meta-analysis). | 5-6 |
| Data collection process | 10 | Describe the data collection process, including whether extraction was performed independently and in duplicate, the use of standardized forms, and any processes used to obtain or confirm missing information from trial authors or supplementary sources. | 5-6 |
| Data items | 11 | List and define all variables for which data were sought (e.g., participant characteristics, intervention details, comparator characteristics, outcomes, follow-up duration, concomitant treatment, and supervision), and describe any assumptions or simplifications made during intervention coding or outcome selection. | 6 |
| **Geometry of the network** | **S1** | Describe the methods used to examine the geometry of the treatment network and any potential biases related to network structure. This should include how the evidence base was graphically summarized and how features such as a sparse or predominantly star-shaped network were considered in interpretation. | ***7-8*** |
| Risk of bias within individual studies | 12 | Describe the methods used for assessing risk of bias in individual studies, including whether this was undertaken at the study or outcome level, and how this information informed data synthesis and interpretation. | 6 |
| Summary measures | 13 | State the principal summary measures (e.g., mean differences with 95% credible intervals for continuous outcomes). Also describe any additional summary measures, such as treatment rankings, and how summary findings from the network meta-analysis were presented. | 7 |
| Planned methods of analysis | 14 | Describe the methods of handling data and combining results of studies for each network meta-analysis. This should include, but not be limited to:   - *Handling of multi-arm trials;* - *specification of the random-effects model and between-study heterogeneity;* - *prior distributions used in the Bayesian analysis;* - assessment of model convergence and fit. | 5, 7-8 |
| **Assessment of Inconsistency** | **S2** | Describe the statistical methods used to assess inconsistency or incoherence in the treatment network, including comparisons between consistency and inconsistency models where applicable, and any actions taken if important inconsistency was detected. | 8 |
| Risk of bias across studies | 15 | Specify any assessment of risk of bias across studies that may affect the cumulative evidence, such as small-study effects, publication bias, or selective reporting. | **8** |
| Additional analyses | 16 | Describe methods for additional analyses, indicating which were pre-specified. These may include sensitivity analyses, network meta-regression, alternative intervention classifications, alternative prior specifications, or leave-one-out analyses. | ***7-8*** |
| **RESULTS†** |  |  |  |
| Study selection | 17 | Report the numbers of studies screened, assessed for eligibility, and included in the review and network meta-analysis, with reasons for exclusion at each stage, ideally using a flow diagram. | 8-9 |
| **Presentation of network structure** | **S3** | Provide a network graph of the included studies to enable visualization of the treatment network for each analyzed outcome or for the overall network, as appropriate. | ***16-18*** |
| **Summary of network geometry** | **S4** | Provide a brief summary of the network geometry, including the distribution of trials and participants across intervention nodes, the extent to which the network was connected through usual care, any gaps in direct evidence, and implications for interpretation. | ***16-18*** |
| Study characteristics | 18 | For each included study, present the characteristics for which data were extracted (e.g., study size, population, intervention, comparator, follow-up, and outcomes) and provide citations. | 9–14 |
| Risk of bias within studies | 19 | Present data on risk of bias for each included study and, where relevant, for specific outcomes. | 15–16 |
| Results of individual studies | 20 | For all outcomes considered, present for each study the available summary data for each intervention group and the corresponding effect estimates with measures of uncertainty, where applicable. | 11–14 |
| Synthesis of results | 21 | Present the results of each network meta-analysis, including summary effect estimates with 95% credible intervals. Where appropriate, present results versus a common comparator, along with league tables, forest plots, or treatment rankings. | 18–23 |
| **Exploration for inconsistency** | **S5** | Describe the results of inconsistency or incoherence assessments, including model fit comparisons or other relevant diagnostics. | 22; Appendix 9/14 |
| Risk of bias across studies | 22 | Present the results of any assessment of risk of bias across studies for the evidence base under review. | 22–23; Appendix 14 |
| Results of additional analyses | 23 | Present the results of additional analyses, such as sensitivity analyses, network meta-regression, alternative network specifications, or prior sensitivity analyses, where undertaken. | ***23*** |
| **DISCUSSION** |  |  |  |
| Summary of evidence | 24 | Summarise the main findings, including the strength or certainty of evidence for the principal outcomes, and discuss their relevance for clinical nutrition and lifestyle management in psoriasis. | 23-26 |
| Limitations | 25 | Discuss limitations at the study, outcome, and review level, including risk of bias, imprecision, network sparsity, the plausibility of transitivity, and the assessment of inconsistency. Comment on any concerns related to the geometry of the network. | 26 |
| Conclusions | 26 | Provide an overall interpretation of the findings in the context of existing evidence and discuss implications for clinical practice and future research. | 27 |
| **FUNDING** |  |  | 27 |
| Funding | 27 | Describe sources of funding and other support for the review, the role of the funders, and any potential conflicts of interest relevant to the interventions included in the network. |  |

**Appendix 2: Search strategy**

**Table S1.** Search strategy of PubMed

| **#** | **Searches** |  |
| --- | --- | --- |
| 1 | ((((psoriasis[MeSH Terms]) OR (psoriasis[Title/Abstract]) OR (psoriases[Title/Abstract]) OR (palmoplantar pustulosis[Title/Abstract]) OR (pustular psoriasis[Title/Abstract])))) |  |
| 2 | ((((psoriatic arthritis[MeSH Terms]) OR (psoriatic arthritis[Title/Abstract]) OR (psoriatic arthropathy[Title/Abstract]) OR (arthritic psoriasis[Title/Abstract])))) |  |
| 3 | #1 OR #2 |  |
| 4 | ((((weight loss[MeSH Terms]) OR (weight loss[Title/Abstract]) OR (weight reduction[Title/Abstract]) OR (weight control[Title/Abstract]) OR (diet therapy[MeSH Terms]) OR (diet[Title/Abstract]) OR (low calorie diet[Title/Abstract]) OR (energy restriction[Title/Abstract]) OR (very low energy diet[Title/Abstract]) OR (diet intervention[Title/Abstract])))) |  |
| 5 | ((((exercise[MeSH Terms]) OR (exercise therapy[MeSH Terms]) OR (physical activity[Title/Abstract]) OR (exercise[Title/Abstract]) OR (exercise training[Title/Abstract]) OR (aerobic exercise[Title/Abstract]) OR (resistance training[Title/Abstract]) OR (strength training[Title/Abstract]) OR (endurance training[Title/Abstract]) OR (physical exercise[Title/Abstract])))) |  |
| 6 | #4 OR #5 |  |
| 7 | #3 AND #6 |  |
| 8 | ((((meta-analysis[Publication Type]) OR (systematic review[Publication Type]) OR (meta-analysis[Title/Abstract]) OR (systematic review[Title/Abstract])))) |  |
| 9 | #7 NOT #8 |  |
| 10 | ((((randomized controlled trial[Publication Type]) OR (controlled clinical trial[Publication Type]) OR (clinical trials as topic[MeSH Terms]) OR (randomized[Title/Abstract]) OR (randomized[Title/Abstract]) OR (randomly[Title/Abstract]) OR (trial[Title/Abstract]) OR (placebo[Title/Abstract])))) |  |
| 11 | #9 AND #10 |  |

**Table S2.** Search strategy of Web of Science

| **#** | **Searches** |
| --- | --- |
| 1 | (((((((TS=(psoriasis)) OR TS=(psoriases)) OR TS=("palmoplantar pustulosis")) OR TS=("pustular psoriasis")))) |
| 2 | (((((TS=("psoriatic arthritis")) OR TS=("psoriatic arthropathy")) OR TS=("arthritic psoriasis")))) |
| 3 | #1 OR #2 |
| 4 | ((((((((((TS=("weight loss")) OR TS=("weight reduction")) OR TS=("weight control")) OR TS=(diet)) OR TS=("low calorie diet")) OR TS=("energy restriction")) OR TS=("very low energy diet")) OR TS=("diet intervention")))) |
| 5 | (((((((((TS=(exercise)) OR TS=("exercise therapy")) OR TS=("physical activity")) OR TS=("exercise training")) OR TS=("aerobic exercise")) OR TS=("resistance training")) OR TS=("strength training")) OR TS=("endurance training")) OR TS=("physical exercise")))) |
| 6 | #4 OR #5 |
| 7 | #3 AND #6 |
| 8 | (((TS=("meta-analysis")) OR TS=("systematic review"))) |
| 9 | #7 NOT #8 |
| 10 | (((((((TS=(randomized)) OR TS=(randomized)) OR TS=(randomly)) OR TS=(trial)) OR TS=(placebo)))) |
| 11 | #9 AND #10 |

**Table S3.** Search strategy of Embase

| **#** | **Searches** |
| --- | --- |
| 1 | ('psoriasis'/exp OR psoriasis:ti,ab OR psoriases:ti,ab OR 'palmoplantar pustulosis':ti,ab OR 'pustular psoriasis':ti,ab) |
| 2 | ('psoriatic arthritis'/exp OR 'psoriatic arthritis':ti,ab OR 'psoriatic arthropathy':ti,ab OR 'arthritic psoriasis':ti,ab) |
| 3 | #1 OR #2 |
| 4 | ('weight loss'/exp OR 'weight reduction':ti,ab OR 'weight control':ti,ab OR 'diet therapy'/exp OR diet:ti,ab OR 'low calorie diet':ti,ab OR 'energy restriction':ti,ab OR 'very low energy diet':ti,ab OR 'diet intervention':ti,ab) |
| 5 | ('exercise'/exp OR 'exercise therapy'/exp OR 'physical activity':ti,ab OR exercise:ti,ab OR 'exercise training':ti,ab OR 'aerobic exercise':ti,ab OR 'resistance training':ti,ab OR 'strength training':ti,ab OR 'endurance training':ti,ab OR 'physical exercise':ti,ab) |
| 6 | #4 OR #5 |
| 7 | #3 AND #6 |
| 8 | ('meta analysis'/exp OR 'systematic review'/exp OR 'meta analysis':ti,ab OR 'systematic review':ti,ab) |
| 9 | #7 NOT #8 |
| 10 | ('randomized controlled trial'/exp OR 'controlled clinical trial'/exp OR random*:ti,ab OR placebo:ti,ab OR trial:ti,ab) |
| 11 | #9 AND #10 |

**Table S4.** Search strategy of Cochrane Central Register of Controlled Trials

| **#** | **Searches** |
| --- | --- |
| 1 | (psoriasis OR psoriases OR "palmoplantar pustulosis" OR "pustular psoriasis"):ti,ab,kw |
| 2 | ("psoriatic arthritis" OR "psoriatic arthropathy" OR "arthritic psoriasis"):ti,ab,kw |
| 3 | #1 OR #2 |
| 4 | ("weight loss" OR "weight reduction" OR "weight control" OR diet OR "low calorie diet" OR "energy restriction" OR "very low energy diet" OR "diet intervention"):ti,ab,kw |
| 5 | (exercise OR "exercise therapy" OR "physical activity" OR "exercise training" OR "aerobic exercise" OR "resistance training" OR "strength training" OR "endurance training" OR "physical exercise"):ti,ab,kw |
| 6 | #4 OR #5 |
| 7 | #3 AND #6 |

**Table S5.** Search strategy of CNKI

| **#** | **Searches** |
| --- | --- |
| 1 | Subject = (psoriasis OR psoriases OR "psoriasis vulgaris" OR "palmoplantar pustulosis" OR "pustular psoriasis" OR "guttate psoriasis") |
| 2 | Subject = ("psoriatic arthritis" OR "psoriatic arthropathy" OR "psoriasis arthropathica") |
| 3 | #1 OR #2 |
| 4 | Subject = ("weight loss" OR "weight reduction" OR "weight control" OR "weight management" OR "diet therapy" OR "dietary intervention" OR "low-calorie diet" OR "very low calorie diet" OR "energy restriction" OR "caloric restriction") |
| 5 | Subject = (exercise OR "exercise therapy" OR "physical activity" OR "exercise training" OR "aerobic exercise" OR "aerobic training" OR "resistance training" OR "resistance exercise" OR "strength training" OR "endurance training" OR "physical exercise") |
| 6 | #4 OR #5 |
| 7 | #3 AND #6 |
| 8 | Subject = ("systematic review" OR "meta-analysis") |
| 9 | #7 NOT #8 |
| 10 | Subject = ("randomized controlled trial" OR "controlled clinical trial" OR "clinical trial" OR randomized OR randomized OR randomly OR trial OR placebo) |
| 11 | #9 AND #10 |

**Table S6.** Search strategy of VIP Database

| **#** | **Searches** |
| --- | --- |
| 1 | Subject = (psoriasis OR psoriases OR "psoriasis vulgaris" OR "palmoplantar pustulosis" OR "pustular psoriasis" OR "guttate psoriasis") |
| 2 | Subject = ("psoriatic arthritis" OR "psoriatic arthropathy" OR "psoriasis arthropathica") |
| 3 | #1 OR #2 |
| 4 | Subject = ("weight loss" OR "weight reduction" OR "weight control" OR "weight management" OR "diet therapy" OR "dietary intervention" OR "low-calorie diet" OR "very low calorie diet" OR "energy restriction" OR "caloric restriction") |
| 5 | Subject = (exercise OR "exercise therapy" OR "physical activity" OR "exercise training" OR "aerobic exercise" OR "aerobic training" OR "resistance training" OR "resistance exercise" OR "strength training" OR "endurance training" OR "physical exercise") |
| 6 | #4 OR #5 |
| 7 | #3 AND #6 |
| 8 | Subject = ("systematic review" OR "meta-analysis") |
| 9 | #7 NOT #8 |
| 10 | Subject = ("randomized controlled trial" OR "controlled clinical trial" OR "clinical trial" OR randomized OR randomized OR randomly OR trial OR placebo) |
| 11 | #9 AND #10 |

**Table S7.** Search strategy of Wanfang Data

| **#** | **Searches** |
| --- | --- |
| 1 | Subject = (psoriasis OR psoriases OR "psoriasis vulgaris" OR "palmoplantar pustulosis" OR "pustular psoriasis" OR "guttate psoriasis") |
| 2 | Subject = ("psoriatic arthritis" OR "psoriatic arthropathy" OR "psoriasis arthropathica") |
| 3 | #1 OR #2 |
| 4 | Subject = ("weight loss" OR "weight reduction" OR "weight control" OR "weight management" OR "diet therapy" OR "dietary intervention" OR "low-calorie diet" OR "very low calorie diet" OR "energy restriction" OR "caloric restriction") |
| 5 | Subject = (exercise OR "exercise therapy" OR "physical activity" OR "exercise training" OR "aerobic exercise" OR "aerobic training" OR "resistance training" OR "resistance exercise" OR "strength training" OR "endurance training" OR "physical exercise") |
| 6 | #4 OR #5 |
| 7 | #3 AND #6 |
| 8 | Subject = ("systematic review" OR "meta-analysis") |
| 9 | #7 NOT #8 |
| 10 | Subject = ("randomized controlled trial" OR "controlled clinical trial" OR "clinical trial" OR randomized OR randomized OR randomly OR trial OR placebo) |
| 11 | #9 AND #10 |

**Table S8.** Search strategy of Chinese Biomedical Literature Database

| **#** | **Searches** |
| --- | --- |
| 1 | Subject = (psoriasis OR psoriases OR "palmoplantar pustulosis" OR "pustular psoriasis" OR "guttate psoriasis") |
| 2 | Subject = ("psoriatic arthritis" OR "psoriatic arthropathy" OR "psoriasis arthropathica") |
| 3 | #1 OR #2 |
| 4 | Subject = ("weight loss" OR "weight reduction" OR "weight control" OR "weight management" OR "diet therapy" OR "dietary intervention" OR "low-calorie diet" OR "very low energy diet" OR "energy restriction" OR "caloric restriction") |
| 5 | Subject = (exercise OR "exercise therapy" OR "physical activity" OR "exercise training" OR "aerobic exercise" OR "resistance training" OR "strength training" OR "endurance training") |
| 6 | #4 OR #5 |
| 7 | #3 AND #6 |
| 8 | Subject = ("randomized controlled trial" OR "controlled clinical trial" OR "clinical trial" OR "randomized trial" OR "random allocation" OR placebo) |
| 9 | #7 AND #8 |

**Appendix 3: Pre-specified classification framework for intervention nodes included in the network meta-analysis**

| **Node** | **Full name** | **Definition** | **Core intervention target** | **Representative examples** | **Decision rules** |
| --- | --- | --- | --- | --- | --- |
| AE | Aerobic exercise | Structured aerobic activity delivered as the principal intervention component | Improve cardiorespiratory fitness and increase energy expenditure | Walking, cycling, treadmill training, supervised aerobic sessions | Classified as AE when aerobic exercise was the main active component and no formal dietary intervention was co-prescribed. |
| D+AE | Diet plus aerobic exercise | Combined dietary restriction and aerobic exercise delivered within the same intervention program | Reduce adiposity through both caloric restriction and aerobic energy expenditure | Diet plus walking, calorie restriction plus supervised aerobic training | Classified as D+AE when both diet and aerobic exercise were explicitly prescribed and both were intended to contribute to treatment effect. |
| LED | Low-energy diet | Dietary intervention primarily based on caloric restriction | Reduce body weight and adiposity through energy restriction | Low-energy diet, very-low-energy diet, hypocaloric diet | Classified as LED when dietary energy restriction was the principal active component and no structured exercise program was co-prescribed. |
| UC | Usual Care | Routine care or minimal advice without any structured active dietary or exercise intervention. | Routine care or minimal lifestyle advice without structured active intervention | Standard care, routine follow-up, brief advice, minimal education | Comparator arms were classified as UC when they did not include a structured dietary or exercise program as an active intervention component. |

**Appendix 4: Deviations from the registered protocol**

| **Protocol element** | **Registered in PROSPERO** | **Final review / manuscript** | **Rationale for deviation** |
| --- | --- | --- | --- |
| Review focus / objective | The registered review broadly aimed to evaluate weight-loss and exercise interventions for psoriasis, with particular emphasis on psoriasis severity, symptoms, and function. | The final review focused more specifically on adiposity-targeting lifestyle interventions in people with psoriasis and overweight or obesity, while also examining psoriasis-specific outcomes. | This refinement was made to better align the review question with the structure of the available randomized evidence and to provide a more clinically focused comparison of interventions targeting adiposity in psoriasis. The change was implemented before quantitative synthesis. |
| Population eligibility | Adults with psoriasis were eligible; studies primarily enrolling patients with psoriatic arthritis were to be excluded unless psoriasis-specific data were separately extractable. | Studies involving participants with psoriatic arthritis were also considered eligible when they were judged to fall within the broader psoriatic disease spectrum and met the remaining eligibility criteria. | This refinement was made to avoid exclusion of potentially relevant randomized evidence from populations with overlapping psoriatic disease features, while maintaining clinical relevance to psoriasis management. The change was implemented before study selection was completed. |
| Outcome hierarchy | Primary outcomes were limited to Psoriasis Area and Severity Index (PASI) and Dermatology Life Quality Index (DLQI); no additional outcomes were specified. | Adiposity-related outcomes, namely body mass index (BMI), body weight, and waist circumference, were specified as primary outcomes. PASI, DLQI, and safety outcomes were analyzed as secondary outcomes. | This refinement was made to better align the outcome framework with the final review objective of evaluating adiposity-targeting lifestyle interventions in psoriasis. The change was implemented before quantitative synthesis. |
| Statistical framework | A frequentist network meta-analysis with 95% confidence intervals and SUCRA was planned. | A Bayesian random-effects network meta-analysis with 95% credible intervals was used as the primary analytical approach. Frequentist analyses were additionally undertaken as supplementary robustness analyses. | This refinement was made to better accommodate the sparse and predominantly star-shaped network structure and to allow probabilistic estimation of relative effects, while retaining a supplementary frequentist assessment of robustness. |
| Intervention classification | A broad range of weight-loss and exercise interventions was anticipated. | Eligible interventions were grouped into four final nodes: low-energy diet (LED), diet plus aerobic or walking exercise (D+AE), aerobic exercise alone (AE), and usual care (UC). | This refinement was made to ensure clinically interpretable and analytically connected treatment nodes for network meta-analysis, based on the characteristics of the available trials. |
| Search strategy | The registry entry did not provide a full reproducible search strategy and indicated limited additional search methods. | The final review searched multiple English- and Chinese-language databases, supplemented by hand-searching of reference lists. Full reproducible search strategies were reported in the supplementary materials. | This refinement was made to improve the completeness, transparency, and reproducibility of the evidence search. |
| Data clarification procedures | Registry wording on retrieval of additional information and author contact was limited and partly inconsistent. | Trial registrations, study protocols, supplementary materials, and author contact were used where necessary to clarify incompletely reported information. | This refinement was made to improve data completeness and reduce uncertainty during data extraction and intervention coding. |

**Appendix 5: Pre-specified imputation hierarchy for missing variance data**

In accordance with the Cochrane Handbook for Systematic Reviews of Interventions, when standard deviations (SDs) or standard errors (SEs) for continuous outcomes were not directly reported, a pre-specified hierarchy of algebraic derivation and imputation was applied. First, when exact P values, t statistics, or 95% confidence intervals (CIs) were available, missing SDs or SEs were derived using standard formulae. Second, when alternative dispersion measures such as interquartile ranges or total ranges were reported, SDs were estimated using established statistical methods where appropriate. Third, for studies reporting baseline and follow-up means and SDs but not the SD of the change score, the missing change-score SD was calculated using the standard formula:

$${SD}_{change} = \sqrt{{{SD}^{2}}_{baseline} + {{SD}^{2}}_{final} - \left( 2\times r \times{SD}_{baseline}\times{SD}_{final} \right)}$$

where r represents the within-group correlation coefficient. When possible, r was derived from included studies reporting complete variance data for the same outcome; otherwise, a default value of 0.5 was assumed. Finally, when variance data could not be directly derived using the above methods, SDs were imputed from studies reporting the same outcome on a comparable scale and with a similar intervention context. The potential influence of studies requiring imputed variance data was further examined in leave-one-out sensitivity analyses.

**Appendix 6: Full text articles excluded after full text review with reasons for exclusion**

| **No.** | **Study** | **Full Citation** | **Reason for Exclusion** |
| --- | --- | --- | --- |
|  | **Not a randomized controlled trial (n = 15)** | | |
| 1 | Zhao et al. | Zhao YJ, Li L, Li WS, Sun P, カルデナス暁東, Cui HZ. Best evidence summary of non-pharmacological weight management in overweight/obese patients with psoriasis. Military Nursing. 2025;42(2):103-107. | Not a randomized controlled trial (evidence summary). |
| 2 | Zhang | Zhang BY. Analysis of diagnostic and treatment characteristics of hospitalized patients with psoriatic arthritis. Liaoning University of Traditional Chinese Medicine; 2025. | Not a randomized controlled trial (thesis/dissertation). |
| 3 | Yetişir et al. | Yetişir A, Sariyildiz A, Coskun Benlidayi I, Özbek S. Type D personality and social inhibition: drivers of cardiovascular risk and reduced physical activity in psoriatic arthritis. Clin Rheumatol. 2025;44(3):1163-1171. doi:10.1007/s10067-025-07312-3 | Not a randomized controlled trial (observational study). |
| 4 | Xiao et al. | Xiao Y, Yang S, Zhang Y, et al. The impact of lifestyle factors on cardiometabolic comorbidities and mortality of psoriatic diseases: A multistate model analysis. J Am Acad Dermatol. 2025;93(3):715-723. doi:10.1016/j.jaad.2025.05.1395 | Not a randomized controlled trial (observational study). |
| 5 | Sahu et al. | Sahu S, Jain P, Deshmukh R, Samal PK, Satapathy T, Ajazuddin. Psoriatic arthritis in transition: cytokine signaling, immune dysregulation, and therapeutic innovation. Inflammopharmacology. 2025;33(8):4275-4290. doi:10.1007/s10787-025-01865-0 | Not a randomized controlled trial (review article). |
| 6 | Näslund-Koch et al. | Näslund-Koch C, Kvist-Hansen A, Bojesen SE, Skov L, Kobylecki CJ, Vedel-Krogh S. Low-grade systemic inflammation is associated with risk of psoriasis in a general population study of more than 100 000 individuals. Br J Dermatol. 2025;193(2):250-258. doi:10.1093/bjd/ljaf147 | Not a randomized controlled trial (observational study). |
| 7 | McGagh et al. | McGagh D, Song K, Yuan H, et al. Digital health technologies to strengthen patient-centred outcome assessment in clinical trials in inflammatory arthritis. Lancet Rheumatol. 2025;7(1):e55-e63. doi:10.1016/S2665-9913(24)00186-3 | Not a randomized controlled trial (review/methodological paper). |
| 8 | Kuszmiersz et al. | Kuszmiersz P, Guła Z, Strach M, et al. Work productivity and activity impairment in patients with rheumatoid arthritis, psoriatic arthritis, and axial spondyloarthritis: a comparative analysis from the POLNOR-Rheuma cohort. Rheumatol Int. 2025;45(5):96. doi:10.1007/s00296-025-05851-1 | Not a randomized controlled trial (observational study). |
| 9 | Kaerts et al. | Kaerts M, de Vlam K, Lories R, Neerinckx B, Swinnen TW. Impaired cardiorespiratory fitness in psoriatic arthritis: insights from cardiopulmonary exercise testing. RMD Open. 2025;11(4). doi:10.1136/rmdopen-2025-006110 | Not a randomized controlled trial (observational study). |
| 10 | Guła et al. | Guła Z, Krzyściak W, Kuszmiersz P, Bystrowska B, Korkosz M. Higher serotonin levels among patients with rheumatoid arthritis, psoriatic arthritis, and axial spondyloarthritis compared to healthy controls assessed by liquid chromatography-tandem mass spectrometry (LC-MS). Rheumatol Int. 2025;45(1):17. doi:10.1007/s00296-024-05769-0 | Not a randomized controlled trial (observational study). |
| 11 | Elsehrawy et al. | Elsehrawy GG, Courvoisier DS, Deman E, et al. Topography of entheseal lesions on ultrasound: a comparative study between axial spondyloarthritis, psoriatic arthritis, and healthy controls. RMD Open. 2025;11(4). doi:10.1136/rmdopen-2025-006088 | Not a randomized controlled trial (observational study). |
| 12 | Coates et al. | Coates LC, Bukhari M, Chan A, et al. Enhancing current guidance for psoriatic arthritis and its comorbidities: recommendations from an expert consensus panel. Rheumatology (Oxford). 2025;64(2):561-573. doi:10.1093/rheumatology/keae172 | Not a randomized controlled trial (consensus/recommendation paper). |
| 13 | Cagnotto et al. | Cagnotto G, Bruschettini M, Stróżyk A, Scirè CA, Compagno M. Tumor necrosis factor (TNF) inhibitors for psoriatic arthritis. Cochrane Database Syst Rev. 2025;2(2):CD013614. doi:10.1002/14651858.CD013614.pub2 | Not a randomized controlled trial (systematic review). |
| 14 | Huang | Huang SQ. Early screening and clinical analysis of arthritis in patients with plaque psoriasis. Dalian Medical University; 2024. | Not a randomized controlled trial (thesis/dissertation). |
| 15 | Guo | Guo H. Changes in left ventricular function in patients with psoriatic arthritis evaluated by quantitative myocardial motion analysis. Jinzhou Medical University; 2024. | Not a randomized controlled trial (thesis/dissertation). |
|  | **Intervention did not meet prespecified definitions or intensity thresholds for dietary or exercise interventions (n = 12)** | | |
| 16 | Nicolau et al. | Nicolau J, Nadal A, Sanchís P, et al. Effects of six months treatment with liraglutide among patients with psoriasis and obesity, beyond metabolic control? Med Clin (Barc). 2025;164(11):106941. doi:10.1016/j.medcli.2025.106941 | Intervention did not meet prespecified eligibility criteria (pharmacological intervention). |
| 17 | Attar et al. | Attar JA, von Martial S, Troost K, et al. Impact of a dermatological rehabilitation program on cardiovascular risks of psoriasis patients. J Dtsch Dermatol Ges. 2025;23(2):161-171. doi:10.1111/ddg.15585 | Intervention did not meet prespecified eligibility criteria (non-eligible rehabilitation program). |
| 18 | Li | Li LX. Clinical characteristics of psoriatic arthritis and analysis of predictive factors for early psoriatic arthritis. Shanxi Medical University; 2024. | Intervention did not meet prespecified eligibility criteria (clinical characterization study rather than eligible dietary/exercise intervention). |
| 19 | Li | Li JS. Exploration of patterns of joint involvement in psoriatic arthritis and validation of a simplified assessment tool. Central South University; 2024. | Intervention did not meet prespecified eligibility criteria (assessment/validation study rather than eligible dietary/exercise intervention). |
| 20 | Zhang | Zhang YF. What should patients know about dietary restrictions in psoriasis? Health Care Garden. 2024;(4):40-41. | Intervention did not meet prespecified eligibility criteria (non-eligible article on dietary advice). |
| 21 | Tournadre and Beauger | Tournadre A, Beauger M. Weight loss affects disease activity and treatment response in inflammatory rheumatic diseases. Joint Bone Spine. 2024;91(3):105647. doi:10.1016/j.jbspin.2023.105647 | Intervention did not meet prespecified eligibility criteria (review/commentary rather than eligible trial). |
| 22 | Talotta et al. | Talotta R, Aiello MR, Restuccia R, Magaudda L. Non-Pharmacological Interventions for Treating Psoriatic Arthritis. Altern Ther Health Med. 2024;30(3):36-43. | Intervention did not meet prespecified eligibility criteria (review article rather than eligible trial). |
| 23 | Laguardia et al. | Laguardia F, Owczarczyk-Saczonek A, Maggi P. The role of smoking in psoriasis. Postepy Dermatol Alergol. 2024;41(2):143-148. doi:10.5114/ada.2023.133582 | Intervention did not meet prespecified eligibility criteria (lifestyle exposure rather than structured intervention). |
| 24 | Katsimbri et al. | Katsimbri P, Grivas A, Papadavid E, et al. Mediterranean diet and exercise are associated with better disease control in psoriatic arthritis. Clin Rheumatol. 2024;43(9):2877-2887. doi:10.1007/s10067-024-07080-6 | Intervention did not meet prespecified eligibility criteria (lifestyle exposure rather than structured intervention). |
| 25 | Fedorchenko et al. | Fedorchenko Y, Mahmudov K, Abenov Z, Zimba O, Yessirkepov M. Rehabilitation of patients with inflammatory rheumatic diseases and comorbidities: unmet needs. Rheumatol Int. 2024;44(4):583-591. doi:10.1007/s00296-023-05529-6 | Intervention did not meet prespecified eligibility criteria (non-eligible rehabilitation paper). |
| 26 | Bellinato et al. | Bellinato F, Maurelli M, Geat D, Girolomoni G, Gisondi P. Managing the Patient with Psoriasis and Metabolic Comorbidities. Am J Clin Dermatol. 2024;25(4):527-540. doi:10.1007/s40257-024-00857-0 | Intervention did not meet prespecified eligibility criteria (management/review article). |
| 27 | Saalbach et al. | Saalbach A, Seitz AT, Kohlmann J, et al. Modulation of Dietary Fatty Acids in an Open-Label Study Improves Psoriasis and Dampens the Inflammatory Activation Status. Nutrients. 2023;15(7). doi:10.3390/nu15071698 | Intervention did not meet prespecified definitions or intensity thresholds for dietary/exercise interventions. |
|  | **Did not report eligible outcomes or provided no extractable data (n = 11)** | | |
| 28 | Chen | Chen J. What should patients do after developing psoriasis? Renren Jiankang. 2024;(3):57. doi:10.20252/j.cnki.rrjk.2024.03.029 | Did not report eligible outcomes or provided no extractable data. |
| 29 | Liu | Liu XM. Value of contrast-enhanced ultrasound and magnetic resonance imaging in the diagnosis of psoriatic arthritis. 2024:1. | Did not report eligible outcomes or provided no extractable data. |
| 30 | Quiles-Tsimaratos et al. | Quiles-Tsimaratos N, Gherardi A, Crochard A, et al. The patient's perspective on the burden of psoriasis: findings based on the ROCQ, an online survey. Eur J Dermatol. 2024;34(1):59-67. doi:10.1684/ejd.2024.4606 | Did not report eligible outcomes or provided no extractable data. |
| 31 | Pattanayak | Pattanayak S. Psycho-neuro-immuno-endocrinology: study of the key factors of disease creation. Explor Anim Med Res. 2024;14(2):164-171. doi:10.52635/EAMR/14.2.164-171 | Did not report eligible outcomes or provided no extractable data. |
| 32 | Gu | Gu YX. Clinical characteristics of psoriatic arthritis and genetic susceptibility based on third-generation sequencing. Sichuan University; 2023. | Did not report eligible outcomes or provided no extractable data. |
| 33 | Truong et al. | Truong K, Joseph J, Li M, Speirs V, Smith A. Exploring the relationship between psoriasis skin clearance and body mass index. Australas J Dermatol. 2023;64:30. doi:10.1111/ajd.14037 | Did not report eligible outcomes or provided no extractable data (conference abstract only). |
| 34 | Sheppard et al. | Sheppard R, Gan WK, Onambele-Pearson GL, Young HS. Developing an aerobic exercise intervention for patients with psoriasis to support lifestyle behaviour change and improve health outcomes. Clin Exp Dermatol. 2023;48(1):5-11. doi:10.1093/ced/llac008 | Did not report eligible outcomes or provided no extractable data (intervention development paper). |
| 35 | Schwarz et al. | Schwarz C, Schwarz M, Schmidbauer V, et al. Diffuse idiopathic skeletal hyperostosis as a cause for dysphagia in a patient with ankylosing spondylitis. Wien Klin Wochenschr. 2023;135(11-12):311-315. doi:10.1007/s00508-023-02205-4 | Did not report eligible outcomes or provided no extractable data. |
| 36 | Queiro et al. | Queiro R, Seoane-Mato D, Laiz A, et al. Confounders contributing to explain the association between sex and disease impact in patients with recent-onset psoriatic arthritis. Clin Exp Rheumatol. 2023;41(1):137-144. doi:10.55563/clinexprheumatol/077ul6 | Did not report eligible outcomes or provided no extractable data. |
| 37 | Pujitha et al. | Pujitha BB, Ramadevi B, Sahithi KN, Ramdas K. Clinicoepidemiological Study of Palmoplantar Dermatoses in Patients Attending Tertiary Care Centre. Eur J Cardiovasc Med. 2023;13(2):1163-1169. | Did not report eligible outcomes or provided no extractable data. |
| 38 | Pavlova et al. | Pavlova NT, Ramasawmy C, Picariello F, Smith C, Moss-Morris R. “I don't know which is the chicken and which is the egg”: A qualitative study of weight loss-related beliefs and behaviours among adults with psoriasis and comorbid obesity. Br J Health Psychol. 2023;28(2):532-551. doi:10.1111/bjhp.12639 | Did not report eligible outcomes or provided no extractable data (qualitative study). |
|  | **Included ineligible populations (n = 4)** | | |
| 39 | Polak et al. | Polak D, Korkosz M, Guła Z. Sleep disorders in rheumatoid arthritis, axial spondyloarthritis and psoriatic arthritis. Rheumatol Int. 2025;45(2):36. doi:10.1007/s00296-024-05780-5 | Included an ineligible population (mixed inflammatory disease population). |
| 40 | Pei et al. | Pei W, Xu L, Zhong H, et al. Clinical features of inflammatory arthritis in daily practice-China's perspective. Clin Rheumatol. 2025;44(3):969-978. doi:10.1007/s10067-024-07262-2 | Included an ineligible population (mixed inflammatory disease population). |
| 41 | Yan | Yan SC. Establishment of a mannan-induced DBA/1 mouse model of psoriatic arthritis and investigation of its mechanisms. Naval Medical University, Chinese People’s Liberation Army; 2024. | Included an ineligible population (animal study). |
| 42 | Chen | Chen YR. Effects of time-restricted feeding on an IMQ-induced mouse model of psoriasis and its underlying mechanisms. Peking Union Medical College; 2023. | Included an ineligible population (animal study). |
|  | **Duplicate publication (n = 2)** | | |
| 43 | Tillett et al. | Tillett W, Coates L, Vis M, et al. Minimal important difference (MID), minimal detectable change (MDC), and disease activity thresholds for two novel composite instruments (3VAS, 4VAS) in patients with psoriatic arthritis: pooled analysis of three phase 3 studies. Ann Rheum Dis. 2023;82:343-344. doi:10.1136/annrheumdis-2023-eular.2237 | Duplicate publication. |
| 44 | Tillett et al. | Tillett W, Coates L, Vis M, et al. Minimal important difference, minimal detectable change and disease activity thresholds for two novel composite instruments, three visual analogue scale/VAS and four visual analogue scale/VAS, in patients with psoriatic arthritis: pooled analysis of three phase III studies. Rheumatology (United Kingdom). 2023;62:ii108-ii109. doi:10.1093/rheumatology/kead104.217 | Duplicate publication. |

**Appendix 7: Risk of bias of randomized clinical trials**

**Figure S7.1:** Overall risk of bias presented as percentage of each risk of bias item across all included studies.

Green = Low risk, Red = High risk, Yellow = Some concerns.

**Figure S7.2:** Study-level risk of bias assessment using the Cochrane RoB 2.0 tool for each included trial.

**Table S7.1:** Study level risk of bias assessment using Cochrane risk of bias tool 2.0 for assessing risk of bias of randomized clinical trials.

| **Study** | **Randomization process** | **Deviations from intended interventions** | **Missing outcome data** | **Measurement of the outcome** | **Selection of the reported result** | **overall** |
| --- | --- | --- | --- | --- | --- | --- |
| Ismail 2024 | some concerns | some concerns | Low | Low | Low | some concerns |
| Ismail 2023 | some concerns | some concerns | Low | Low | Low | some concerns |
| Landgren 2025 | Low | Low | Low | Low | Low | Low |
| Diaz 2023 | Low | Low | Low | Low | Low | Low |
| Leite 2022 | Low | Low | Low | Low | Low | Low |
| Naldi 2014 | some concerns | some concerns | Low | Low | some concerns | some concerns |
| Jensen 2014 | some concerns | some concerns | Low | Low | some concerns | some concerns |
| Al-Mutairi 2014 | some concerns | some concerns | Low | Low | some concerns | some concerns |
| Jensen 2013 | some concerns | some concerns | Low | Low | Low | some concerns |

**Appendix 8. Data extraction framework, variable definitions, and data handling rules for the network meta-analysis**

Table S8.1: Data extraction framework

| **Domain** | **Variable** | **Description** |
| --- | --- | --- |
| **Study characteristics** | Study ID | First author and year used as unique identifier |
|  | Country | Country where the study was conducted |
|  | Study design | Randomized controlled trial design (parallel or multi-arm) |
|  | Sample size | Number of participants in each study arm |
| **Participant characteristics** | Age | Mean age of participants in each arm |
|  | Sex distribution | Percentage of female participants |
|  | Body mass index | Baseline BMI reported as mean ± SD |
|  | Comorbidities | Reported comorbid conditions (e.g. metabolic syndrome, diabetes, psoriatic arthritis) |
| **Intervention characteristics** | Intervention type | Classified according to pre-specified treatment nodes |
|  | Intervention duration | Duration of intervention in weeks |
|  | Intervention components | Detailed description of diet and/or exercise program |
|  | Co-interventions | Concurrent treatments applied equally across study arms |
| **Comparator characteristics** | Comparator type | Usual care, waitlist, or alternative comparator |
|  | Comparator description | Description of comparator condition |
| **Outcome data** | BMI | Mean and SD at baseline and follow-up |
|  | Body weight | Mean and SD at baseline and follow-up |
|  | Waist circumference | Mean and SD at baseline and follow-up |
|  | PASI | Psoriasis Area and Severity Index |
|  | DLQI | Dermatology Life Quality Index (if reported) |
|  | Safety outcomes | Adverse events and safety-related outcomes, including type, frequency, and severity, as reported in the original studies |
| **Risk of bias** | RoB domains | Domain-level assessment using RoB 2 |
|  | Overall judgement | Overall risk-of-bias classification |

Table S8.2: Data coding and handling rules

| **Rule category** | **Specification** |
| --- | --- |
| Continuous outcomes | Extracted as mean and standard deviation; endpoint values used when change scores were unavailable |
| Unit harmonization | Outcomes converted to a common scale where required |
| Multi-arm trials | All relevant arms included; correlations handled within NMA model |
| Missing data | SD calculated from available statistics when possible; otherwise excluded from that outcome |
| Intervention classification | Based on pre-specified rules in **Appendix 3** |
| Co-interventions | Included if balanced across arms; explored in sensitivity analyses |
| Outcome time point | Closest to end of intervention selected |
| Data extraction process | Performed independently by two reviewers |
| Intention-to-treat | Prioritized when reported |
| Network inclusion | Only comparisons contributing to connected network included |

**Table S8.3:** Detailed characteristics of comparator conditions across included studies

| **Study** | **Comparator category** | **Detailed comparator condition** | **Background treatment** | **Contact and attention provided to comparator** |
| --- | --- | --- | --- | --- |
| Ismail 2024 | Waitlist control | Participants were instructed not to modify their usual lifestyle habits and received no structured dietary or exercise intervention. | Background psoriasis treatment was not clearly reported. | No structured counselling, exercise supervision, or dietary monitoring was reported. |
| Ismail 2023 | Pharmacotherapy-only control | Participants received immunosuppressive drugs without the low-calorie diet or walking programme. | Immunosuppressive therapy was provided to both groups. | No structured lifestyle counselling or monitoring was reported for the comparator group. |
| Landgren 2025 | Active matched comparator | Obese participants without psoriatic arthritis received the same VLED programme as the PsA group, including 640 kcal/day for 12–16 weeks and subsequent food reintroduction. | Usual medical care continued as required. | Both groups received dietary advice, physical activity support, and follow-up at the obesity centre for 12 months. |
| Diaz 2023 | No-exercise control | Participants received no structured aerobic exercise programme and no prescribed dietary intervention. | Participants used topical psoriasis treatment; systemic antipsoriatic therapy was excluded. | Dietary intake and study outcomes were assessed, but no exercise supervision or lifestyle counselling was provided. |
| Leite 2022 | Placebo plus habitual diet | Participants received soybean-oil placebo capsules and were instructed to maintain their habitual diet without caloric restriction. | PsA medications and physical activity were required to remain stable throughout the study. | Participants received monthly monitoring for supplementation and adherence, but no hypocaloric dietary counselling during the intervention. |
| Naldi 2014 | Informational counselling control | Participants received a single 15-min session at baseline describing the potential benefits of weight reduction for psoriasis control. | Systemic psoriasis treatment continued according to local guidelines and could be modified for safety or substantial weight change. | No structured dietary or exercise programme was provided; outcome assessments were conducted during follow-up. |
| Al-Mutairi 2014 | Normal-diet control | Participants continued their normal diet without prescribed caloric restriction. | Both groups continued biologic therapy for psoriasis for 24 weeks. | Clinical assessments were performed every 4 weeks; no structured dietary counselling was reported for the comparator group. |
| Jensen 2013 | Attention-matched healthy-diet control | Participants were instructed to consume ordinary healthy foods according to national dietary guidelines. | Antipsoriatic treatment was required to remain stable, and participants were instructed not to change physical activity or tobacco use. | The comparator group attended the same eight biweekly group sessions with the same dietitian and received dietary advice and encouragement. |
| Jensen 2014 | Attention-matched healthy-diet control | Participants were instructed to consume ordinary healthy foods according to national dietary guidelines. | Antipsoriatic treatment was required to remain stable, and participants were instructed not to change physical activity or tobacco use. | The comparator group attended the same eight biweekly group sessions with the same dietitian and received dietary advice and encouragement. |

**Appendix 9: Bayesian prior specification, MCMC settings, convergence diagnostics, heterogeneity, and model fit**

Bayesian random-effects network meta-analysis models were fitted for BMI, body weight, waist circumference, and PASI. Weakly informative priors were used for treatment effects, baseline effects, and between-study heterogeneity. MCMC settings and prior specifications are summarized in Table S9.1. Model convergence was assessed using trace plots, posterior density plots, the potential scale reduction factor (R-hat), and effective sample size (ESS). Across the four outcome-specific models, convergence was satisfactory, with R-hat values close to 1.00 and adequate effective sample sizes, supporting stable posterior estimation. Between-study heterogeneity is summarized by the posterior mean of τ and its 95% credible interval (CrI), and model fit was assessed by comparing fixed- and random-effects models using the deviance information criterion (DIC).

**Table S9.1:** Prior specification and MCMC settings for the Bayesian network meta-analysis models

| **Item** | **Specification** |
| --- | --- |
| Model | Bayesian random-effects consistency network meta-analysis |
| Outcomes | BMI, body weight, waist circumference, PASI |
| Effect measure | Mean difference (MD) |
| Software | R, multinma package |
| Prior for relative treatment effects | Normal(0, 10) |
| Prior for study-specific baseline effects | Normal(0, 100) |
| Prior for between-study heterogeneity | Half-Normal(0, 5) |
| Number of chains | 4 |
| Total iterations per chain | 2000 |
| Burn-in iterations | 1000 |
| Thinning interval | 1 |
| Convergence diagnostics | Trace plots, posterior density plots, R-hat, Bulk ESS, Tail ESS |

****Abbreviations:**** ESS, effective sample size; MD, mean difference.

**Table S9.2:** Convergence diagnostics across outcome-specific Bayesian network meta-analysis models

| **Outcome** | **R-hat range** | **Bulk ESS range** | **Tail ESS range** | **τ, mean (95% CrI)** |
| --- | --- | --- | --- | --- |
| BMI | 1.00–1.01 | 1428–6236 | 1802–3306 | 1.87 (0.49 to 4.27) |
| Body weight | 1.00–1.00 | 1210–4381 | 1119–3490 | 4.97 (0.87 to 10.20) |
| Waist circumference | 1.00–1.00 | 1614–4673 | 1915–3443 | 5.08 (2.21 to 9.58) |
| PASI | 1.00–1.00 | 1371–5063 | 1558–3501 | 2.77 (0.96 to 6.65) |

****Abbreviations:**** CrI, credible interval; ESS, effective sample size; R-hat, potential scale reduction factor; τ, between-study heterogeneity standard deviation.

**Table S9.3: Comparison of fixed- and random-effects Bayesian network meta-analysis models**

| **Outcome** | **Fixed-effects DIC** | **Random-effects DIC** | **ΔDIC (fixed − random)** | **Model used in the primary analysis** |
| --- | --- | --- | --- | --- |
| BMI | 36.9 | 35.8 | 1.1 | Random-effects |
| Body weight | 20.7 | 20.2 | 0.5 | Random-effects |
| Waist circumference | 24.1 | 23.7 | 0.4 | Random-effects |
| PASI | 32.71 | 19.25 | 13.46 | Random-effects |

****Abbreviations:**** DIC, deviance information criterion. Lower DIC values indicate better model fit after accounting for model complexity. Differences in DIC greater than 3 were considered meaningful. For BMI, body weight, and waist circumference, the fixed- and random-effects models showed similar fit. The prespecified random-effects model was retained for the primary analyses to account for potential between-study heterogeneity and to maintain a consistent analytical framework across outcomes. For PASI, the random-effects model showed meaningfully better fit.

**Appendix 10: Bayesian network meta-analysis results, SUCRA, and cumulative ranking probability plots**

**Table S10.1:** BMI

| Comparison | MD (95% CrI) |
| --- | --- |
| AE vs UC | −2.31 (−6.49 to 1.96) |
| D+AE vs UC | −2.20 (−4.81 to 0.39) |
| LED vs UC | −2.89 (−4.78 to −0.73) |

**Table S10.2:** Body weight

| Comparison | MD (95% CrI) |
| --- | --- |
| D+AE vs UC | 0.56 (−9.15 to 9.99) |
| LED vs UC | −7.97 (−13.07 to −0.81) |

**Table S10.3:** Waist circumference

| Comparison | MD (95% CrI) |
| --- | --- |
| D+AE vs UC | −3.54 (−10.18 to 2.57) |
| LED vs UC | −3.41 (−9.59 to 3.17) |

**Table S10.4:** PASI

| Comparison | MD (95% CrI) |
| --- | --- |
| D+AE vs UC | −2.48 (−6.26 to 1.46) |
| LED vs UC | −0.92 (−5.70 to 3.41) |

**Figure S10.1:**

**Cumulative ranking probability plots for the effects of lifestyle interventions on body mass index (BMI).** A larger area under the cumulative ranking curve indicates a higher probability that the intervention is among the most effective for reducing BMI.

****Table S10.5:** SUCRA results for lifestyle interventions on body mass index (BMI).**

| **Intervention** | **Rank ≤1** | **Rank ≤2** | **Rank ≤3** | **Rank ≤4** | **SUCRA** |
| --- | --- | --- | --- | --- | --- |
| UC | 0.00 | 0.01 | 0.16 | 1.00 | 0.06 |
| AE | 0.33 | 0.58 | 0.88 | 1.00 | 0.60 |
| D+AE | 0.20 | 0.58 | 0.96 | 1.00 | 0.58 |
| LED | 0.47 | 0.83 | 0.99 | 1.00 | 0.77 |

### ****Figure S10.2:****

**Cumulative ranking probability plots for the effects of lifestyle interventions on body weight.** A larger area under the cumulative ranking curve indicates a higher probability that the intervention is among the most effective for reducing body weight.

### ****Table S10.6:** SUCRA results for lifestyle interventions on body weight.**

| **Intervention** | **Rank ≤1** | **Rank ≤2** | **Rank ≤3** | **SUCRA** |
| --- | --- | --- | --- | --- |
| UC | 0.01 | 0.57 | 1 | 0.29 |
| D+AE | 0.07 | 0.44 | 1 | 0.26 |
| LED | 0.92 | 0.99 | 1 | 0.95 |

### ****Figure S10.3:****

**Cumulative ranking probability plots for the effects of lifestyle interventions on waist circumference.** A larger area under the cumulative ranking curve indicates a higher probability that the intervention is among the most effective for reducing waist circumference.

### ****Table S10.7:** SUCRA results for lifestyle interventions on waist circumference.**

| **Intervention** | **Rank ≤1** | **Rank ≤2** | **Rank ≤3** | **SUCRA** |
| --- | --- | --- | --- | --- |
| UC | 0.02 | 0.23 | 1 | 0.12 |
| D+AE | 0.5 | 0.89 | 1 | 0.70 |
| LED | 0.5 | 0.88 | 1 | 0.68 |

### ****Figure S10.4:****

**Cumulative ranking probability plots for the effects of lifestyle interventions on Psoriasis Area and Severity Index (PASI).** A larger area under the cumulative ranking curve indicates a higher probability that the intervention is among the most effective for reducing PASI.

### ****Table S10.8:** SUCRA results for lifestyle interventions on Psoriasis Area and Severity Index (PASI).**

| **Intervention** | **Rank ≤1** | **Rank ≤2** | **Rank ≤3** | **SUCRA** |
| --- | --- | --- | --- | --- |
| UC | 0.02 | 0.33 | 1 | 0.18 |
| D+AE | 0.74 | 0.94 | 1 | 0.84 |
| LED | 0.24 | 0.73 | 1 | 0.48 |

### ****Appendix 11: League tables of Bayesian network meta-analysis results for lifestyle interventions in psoriasis****

League tables present posterior mean differences (MDs) and 95% credible intervals (CrIs) for all pairwise comparisons from the Bayesian random-effects network meta-analysis. For BMI, body weight, waist circumference, and PASI, negative values indicate a greater reduction in the outcome and therefore favour the intervention listed in the row.

****Table S11.1:**** Body mass index (BMI)

| AE | -0.17 (-5.02, 4.97) | 0.60 (-4.37, 5.16) | -2.31 (-6.49, 1.96) |
| --- | --- | --- | --- |
| 0.17 (-4.97, 5.02) | D+AE | 0.74 (-2.80, 3.90) | -2.20 (-4.81, 0.39) |
| -0.60 (-5.16, 4.37) | -0.74 (-3.90, 2.80) | LED | -2.89 (-4.78, -0.73) |
| 2.31 (-1.96, 6.49) | 2.20 (-0.39, 4.81) | 2.89 (0.73, 4.78) | UC |

****Table S11.2:**** Body weight

| D+AE | 8.50 (-4.24, 18.73) | 0.56 (-9.15, 9.99) |
| --- | --- | --- |
| -8.50 (-18.73, 4.24) | LED | -7.97 (-13.07, -0.81) |
| -0.56 (-9.99, 9.15) | 7.97 (0.81, 13.07) | UC |

****Table S11.3:**** Waist circumference

| D+AE | -0.13 (-9.34, 8.44) | -3.54 (-10.18, 2.57) |
| --- | --- | --- |
| 0.13 (-8.44, 9.34) | LED | -3.41 (-9.59, 3.17) |
| 3.54 (-2.57, 10.18) | 3.41 (-3.17, 9.59) | UC |

****Table S11.4:**** Psoriasis Area and Severity Index (PASI)

| D+AE | -1.53 (-7.31, 4.55) | -2.48 (-6.26, 1.46) |
| --- | --- | --- |
| 1.53 (-4.55, 7.31) | LED | -0.92 (-5.70, 3.41) |
| 2.48 (-1.46, 6.26) | 0.92 (-3.41, 5.70) | UC |

### ****Appendix 12: CINeMA Assessment****

Certainty of evidence was assessed using the CINeMA framework, which evaluates six domains: within-study bias, reporting bias, indirectness, imprecision, heterogeneity, and incoherence. For each network estimate, judgments were informed by the percentage contribution of direct comparisons to the network and the corresponding risk-of-bias assessments of the contributing studies. Risk of bias for individual studies was assessed using the RoB 2 tool and is presented in Appendix 7. The certainty ratings for each comparison and outcome are reported in the corresponding CINeMA figures and tables in this appendix.

****Within-study bias:**** Judgments for within-study bias were based on the RoB 2 assessments of the studies contributing to each network estimate, taking into account the percentage contribution of each direct comparison within the network. Risk-of-bias assessments for individual studies are presented in Appendix 7, and the contribution of within-study bias to each pairwise network comparison is summarized in the corresponding CINeMA outputs in this appendix.

## ****Figure S12.1:****

Risk-of-bias contribution by intervention group for body mass index (BMI).

## ****Figure S12.2：****

**Risk-of-bias contribution by intervention group for body weight.**

## ****Figure S12.3：****

**Risk-of-bias contribution by intervention group for waist circumference.**

## ****Figure S12.4：****

**Risk-of-bias contribution by intervention group for Psoriasis Area and Severity Index (PASI).**

**Table S12.1:** Transitivity (Indirectness) Assessment

|  | Baseline variable (Mean ± SD) | |
| --- | --- | --- |
| Intervention | Age (year), Mean ± SD | BMI (kg/m²), Mean ± SD |
| AE | 35.00 ± 6.10 | 31.70 ± 3.90 |
| D+AE | 51.58 ± 15.10 | 31.04 ± 5.67 |
| LED | 48.26 ± 9.90 | 31.75 ± 4.81 |
| UC | 49.9 ± 13.0 | 31.9 ± 5.2 |

**Imprecision:** Imprecision was assessed using the CINeMA framework for each network comparison. Judgements were based on the width of the 95% confidence intervals and whether these intervals crossed thresholds of clinical relevance.

**Heterogeneity:** Heterogeneity was assessed within the CINeMA framework by considering whether between-study variability could materially affect the interpretation of the estimated treatment effects. Judgements were informed by the 95% confidence intervals and prediction intervals in relation to clinically important effect sizes, using the same thresholds of clinical relevance as for imprecision.

**Incoherence:** Incoherence was assessed within the CINeMA framework with consideration of the network structure and the consistency between direct and indirect evidence. Because the networks were sparse and predominantly star-shaped, with no closed loops for most outcomes, local inconsistency approaches such as node-splitting could not be performed. Where feasible, global inconsistency was explored by comparing the consistency model with the unrelated mean effects model. However, given the limited network connectivity, the power to detect important incoherence was inherently low. This limitation was considered when rating the certainty of evidence.

**Table S12.2:** CINeMA results for body mass index (BMI)

| Comparison | Within-study bias | Reporting bias | Indirectness | Imprecision | Heterogeneity | Incoherence | Confidence rating |
| --- | --- | --- | --- | --- | --- | --- | --- |
| AE:UC | No concerns | Low risk | No concerns | Major concerns | No concerns | Major concerns | Low |
| D+AE:UC | Some concerns | Low risk | No concerns | No concerns | Major concerns | Major concerns | Low |
| LED:UC | Some concerns | Low risk | No concerns | No concerns | Major concerns | Major concerns | Low |
| AE:D+AE | Some concerns | Low risk | No concerns | Major concerns | No concerns | Major concerns | Low |
| AE:LED | No concerns | Low risk | No concerns | Major concerns | No concerns | Major concerns | Low |
| D+AE:LED | Some concerns | Low risk | No concerns | Major concerns | No concerns | Major concerns | Low |

### ****Table S12.3:** CINeMA results for body weight**

| Comparison | Within-study bias | Reporting bias | Indirectness | Imprecision | Heterogeneity | Incoherence | Confidence rating |
| --- | --- | --- | --- | --- | --- | --- | --- |
| D+AE:UC | Some concerns | Low risk | No concerns | Major concerns | No concerns | Major concerns | Low |
| LED:UC | Some concerns | Low risk | No concerns | No concerns | Major concerns | Major concerns | Low |
| D+AE:LED | Some concerns | Low risk | No concerns | Major concerns | No concerns | Major concerns | Low |

### ****Table S12.4:** CINeMA results for waist circumference**

| Comparison | Within-study bias | Reporting bias | Indirectness | Imprecision | Heterogeneity | Incoherence | Confidence rating |
| --- | --- | --- | --- | --- | --- | --- | --- |
| D+AE:UC | Some concerns | Low risk | No concerns | Major concerns | No concerns | Major concerns | Low |
| LED:UC | Some concerns | Low risk | No concerns | Major concerns | No concerns | Major concerns | Low |
| D+AE:LED | Some concerns | Low risk | No concerns | Major concerns | No concerns | Major concerns | Low |

### ****Table S12.5:** CINeMA results for Psoriasis Area and Severity Index (PASI)**

| Comparison | Within-study bias | Reporting bias | Indirectness | Imprecision | Heterogeneity | Incoherence | Confidence rating |
| --- | --- | --- | --- | --- | --- | --- | --- |
| D+AE:UC | Some concerns | Low risk | No concerns | Major concerns | No concerns | Major concerns | Low |
| LED:UC | Some concerns | Low risk | No concerns | Major concerns | No concerns | Major concerns | Low |
| D+AE:LED | Some concerns | Low risk | No concerns | Major concerns | No concerns | Major concerns | Low |

**Appendix 13: Funnel plots**
These funnel plots were used to visually assess potential small-study effects in studies evaluating lifestyle interventions for psoriasis. The funnel plots cover all trials comparing at least one lifestyle intervention with a control group (e.g., usual care) and analyze the following outcome measures: body mass index (BMI), body weight, waist circumference, and Psoriasis Area and Severity Index (PASI). Comparison-adjusted funnel plots were generated using the **netmeta** package in R, with usual care (UC) specified as the reference comparator (order = "UC"). Even when the number of included studies was insufficient to support a formal test for funnel plot asymmetry, the plots are still presented for visual inspection.

**Figure S13.1:** Funnel Plot for Body Mass Index (BMI).

**Figure S13.2:** Funnel Plot for Body Weight.

**Figure S13.3:** Funnel Plot for Waist Circumference.

**Figure S13.4:** Funnel Plot for Psoriasis Area and Severity Index (PASI).

# ****Appendix 14: Frequentist sensitivity analysis****

**As a sensitivity analysis, all outcome-specific networks were re-estimated under a frequentist random-effects framework using the netmeta package in R. Table S14.1 summarizes heterogeneity and Q statistics from the frequentist analyses, whereas Table S14.2 compares the Bayesian and frequentist effect estimates and ranking metrics across outcomes.**

****Table S14.1:** Heterogeneity and Q statistics from frequentist network meta-analysis.**

| **Outcome** | **Tau²** | **Tau** | **I² (95% CI)** | **Total Q (df, p value)** | **Within-design Q (df, p value)** |
| --- | --- | --- | --- | --- | --- |
| BMI | 1.1395 | 1.0675 | 69.1% (27.1% to 86.9%) | 16.16 (5, 0.0064) | 16.16 (5, 0.0064) |
| Body weight | 22.3938 | 4.7322 | 75.6% (32.8% to 91.2%) | 12.32 (3, 0.0064) | 12.32 (3, 0.0064) |
| Waist circumference | 26.2658 | 5.1250 | 83.1% (61.6% to 92.6%) | 23.73 (4, <0.0001) | 23.73 (4, <0.0001) |
| PASI | 4.45 | 2.11 | 84.3% (60.5% to 93.7%) | 19.08 (3, 0.0003) | 19.08 (3, 0.0003) |

****Abbreviations:**** Tau², between-study heterogeneity variance; Tau, between-study heterogeneity standard deviation; Q, Cochran’s Q statistic.

****Table S14.2:** Comparison of Bayesian and frequentist network meta-analysis results across outcomes.**

| **Outcome** | **Comparison** | **Bayesian MD (95% CrI)** | **Frequentist MD (95% CI)** | **Bayesian SUCRA** | **Frequentist P-score** |
| --- | --- | --- | --- | --- | --- |
| BMI | AE vs UC | -2.31 (-6.49 to 1.96) | -2.40 (-4.91 to 0.11) | 0.60 | 0.5903 |
| BMI | D+AE vs UC | -2.20 (-4.81 to 0.39) | -2.19 (-3.64 to -0.74) | 0.58 | 0.5189 |
| BMI | LED vs UC | -2.89 (-4.78 to -0.73) | -3.41 (-4.76 to -2.05) | 0.77 | 0.8802 |
| Body weight | D+AE vs UC | 0.56 (-9.15 to 9.99) | 0.70 (-9.21 to 10.61) | 0.26 | 0.2521 |
| Body weight | LED vs UC | -7.97 (-13.07 to -0.81) | -8.38 (-14.01 to -2.75) | 0.95 | 0.9695 |
| Waist circumference | D+AE vs UC | -3.54 (-10.18 to 2.57) | -4.05 (-10.59 to 2.49) | 0.70 | 0.7115 |
| Waist circumference | LED vs UC | -3.41 (-9.59 to 3.17) | -3.64 (-10.00 to 2.73) | 0.68 | 0.6665 |
| PASI | D+AE vs UC | -2.48 (-6.26 to 1.46) | -2.57 (-5.27 to 0.13) | 0.84 | 0.8743 |

****Abbreviations:**** CI, confidence interval; CrI, credible interval; MD, mean difference; SUCRA, surface under the cumulative ranking curve.

### ****Appendix 15: Meta-regression analyses****

Pre-specified exploratory network meta-regression analyses were conducted to assess potential effect modification by intervention duration and study-level baseline body mass index (BMI). Both covariates were mean-centred prior to analysis. Model 1 included intervention duration as a single covariate, whereas Model 2 included both intervention duration and baseline BMI. Results are presented as posterior mean regression coefficients and corresponding 95% credible intervals (CrIs). The impact of meta-regression on between-study heterogeneity was examined by comparing τ estimates from the primary network meta-analysis and the meta-regression models.

**Table S15.1:** Bayesian network meta-regression coefficients for prespecified study-level covariates

| **Outcome** | **Model** | **Covariate** | **β (mean)** | **SD** | **95% CrI** | **R-hat** |
| --- | --- | --- | --- | --- | --- | --- |
| BMI | 1 | Duration (weeks) | -0.47 | 6.75 | -13.75 to 12.67 | 1.003 |
| BMI | 2 | Duration (weeks) | -0.25 | 6.75 | -13.12 to 13.50 | 1.010 |
| BMI | 2 | Baseline BMI (kg/m²) | -0.18 | 9.12 | -17.77 to 18.32 | 1.011 |
| Body weight | 1 | Duration (weeks) | 0.13 | 7.80 | -15.39 to 15.08 | 1.005 |
| Body weight | 2 | Duration (weeks) | 0.32 | 7.71 | -15.40 to 14.76 | 1.002 |
| Body weight | 2 | Baseline BMI (kg/m²) | 0.54 | 9.11 | -17.54 to 18.35 | 1.001 |
| Waist circumference | 1 | Duration (weeks) | -0.23 | 6.90 | -12.95 to 14.43 | 1.001 |
| Waist circumference | 2 | Duration (weeks) | 0.30 | 6.56 | -12.69 to 12.80 | 1.002 |
| Waist circumference | 2 | Baseline BMI (kg/m²) | 0.10 | 9.41 | -18.11 to 18.81 | 1.001 |

****Abbreviations:**** BMI, body mass index; CrI, credible interval.

**Table S15.2:** Impact of meta-regression on between-study heterogeneity (τ)

| **Outcome** | **Model** | **τ mean** | **τ median** | **95% CrI** |
| --- | --- | --- | --- | --- |
| BMI | Primary NMA | 1.87 | 1.72 | 0.49 to 4.27 |
| BMI | Model 1 (duration) | 1.79 | 1.60 | 0.24 to 4.41 |
| BMI | Model 2 (duration + BMI) | 1.80 | 1.62 | 0.28 to 4.31 |
| Body weight | Primary NMA | 4.97 | 4.82 | 0.87 to 10.20 |
| Body weight | Model 1 (duration) | 5.02 | 4.82 | 0.80 to 10.30 |
| Body weight | Model 2 (duration + BMI) | 5.02 | 4.82 | 0.84 to 10.20 |
| Waist circumference | Primary NMA | 5.08 | 4.83 | 2.21 to 9.58 |
| Waist circumference | Model 1 (duration) | 5.11 | 4.83 | 2.18 to 9.64 |
| Waist circumference | Model 2 (duration + BMI) | 5.13 | 4.85 | 2.17 to 9.63 |

****Abbreviations:**** CrI, credible interval; τ, between-study heterogeneity standard deviation.

**Appendix 16: Leave-one-out sensitivity analyses**

**Figure S16.1:** Leave-one-out sensitivity analysis for between-study heterogeneity (τ) in BMI

**Table S16.1:** Leave-one-out sensitivity analysis of between-study heterogeneity (τ) for BMI after sequential exclusion of individual studies

| Study removed | τ (mean) | SD | 2.5% CrI | 25% | 50% | 75% | 97.5% CrI | Bulk ESS | Tail ESS | Rhat |
| --- | --- | --- | --- | --- | --- | --- | --- | --- | --- | --- |
| Primary analysis | 1.870 | 1.100 | 0.491 | 1.110 | 1.640 | 2.380 | 4.270 | 860 | 1050 | 1.00 |
| Ismail AMA2023 (1) | 2.310 | 1.320 | 0.381 | 1.420 | 2.060 | 2.940 | 5.660 | 838 | 1183 | 1.00 |
| Ismail AMA2023 (2) | 2.240 | 1.310 | 0.329 | 1.330 | 2.000 | 2.880 | 5.630 | 827 | 904 | 1.00 |
| Diaz AJ 2023 | 1.760 | 1.070 | 0.189 | 1.040 | 1.590 | 2.280 | 4.380 | 873 | 927 | 1.00 |
| Leite BF 2022 | 0.622 | 0.549 | 0.023 | 0.232 | 0.491 | 0.855 | 2.070 | 1096 | 1680 | 1.00 |
| Naldi L 2014 | 1.990 | 1.230 | 0.194 | 1.140 | 1.770 | 2.610 | 5.060 | 863 | 840 | 1.00 |
| Jensen P 2014 | 2.190 | 1.280 | 0.264 | 1.300 | 1.980 | 2.820 | 5.290 | 638 | 633 | 1.00 |
| Al-Mutairi N 2014 | 2.050 | 1.180 | 0.341 | 1.250 | 1.820 | 2.620 | 4.850 | 956 | 1252 | 1.00 |
| Jensen P 2013 | 2.710 | 2.190 | 0.486 | 1.480 | 2.140 | 3.080 | 10.700 | 154 | 27.6 | 1.00 |

**Abbreviations:** CrI, credible interval; ESS, effective sample size; Rhat, potential scale reduction factor.

**Note:** Leave-one-out analyses were performed by iteratively removing one study at a time and refitting the Bayesian random-effects network meta-analysis model. τ represents the estimated between-study heterogeneity. Posterior mean values and corresponding 95% credible intervals are presented. Rhat values close to 1.00 indicate acceptable model convergence.

**Figure S16.2:** Leave-one-out sensitivity analysis for between-study heterogeneity (τ) in body weight

**Table S16.2:** Leave-one-out sensitivity analysis of between-study heterogeneity (τ) for body weight after sequential exclusion of individual studies

| Study removed | τ (mean) | SD | 2.5% CrI | 25% | 50% | 75% | 97.5% CrI | Bulk ESS | Tail ESS | Rhat |
| --- | --- | --- | --- | --- | --- | --- | --- | --- | --- | --- |
| Primary analysis | 4.970 | 2.370 | 0.870 | 3.480 | 4.890 | 6.550 | 10.200 | 1319 | 1170 | 1.00 |
| Leite BF 2022 | 2.850 | 2.160 | 0.109 | 1.220 | 2.400 | 4.020 | 8.130 | 1150 | 1386 | 1.00 |
| Naldi L 2014 | 5.130 | 2.470 | 0.776 | 3.450 | 4.890 | 6.610 | 10.700 | 1034 | 692 | 1.00 |
| Jensen P 2014 | 5.620 | 2.540 | 1.050 | 3.850 | 5.460 | 7.140 | 11.200 | 1332 | 822 | 1.00 |
| Al-Mutairi N 2014 | 4.660 | 2.500 | 0.578 | 2.880 | 4.360 | 6.190 | 10.200 | 1380 | 1148 | 1.00 |
| Jensen P 2013 | 5.890 | 2.460 | 1.760 | 4.160 | 5.660 | 7.340 | 11.500 | 2168 | 1482 | 1.00 |

**Figure S16.3:** Leave-one-out sensitivity analysis for between-study heterogeneity (τ) in waist circumference

**Table S16.3:** Leave-one-out sensitivity analysis of between-study heterogeneity (τ) for Waist circumference after sequential exclusion of individual studies

| Study removed | τ (mean) | SD | 2.5% CrI | 25% | 50% | 75% | 97.5% CrI | Bulk ESS | Tail ESS | Rhat |
| --- | --- | --- | --- | --- | --- | --- | --- | --- | --- | --- |
| Primary analysis | 5.080 | 1.860 | 2.210 | 3.770 | 4.840 | 6.140 | 10.200 | 1829 | 2272 | 1.00 |
| Ismail AMA2023 (study 1) | 4.630 | 2.100 | 1.390 | 3.140 | 4.350 | 5.830 | 9.600 | 1841 | 1540 | 1.00 |
| Ismail AMA2023 (study 2) | 5.340 | 2.130 | 2.040 | 3.840 | 5.050 | 6.500 | 10.200 | 2001 | 1800 | 1.00 |
| Leite BF 2022 | 4.950 | 2.060 | 1.810 | 3.450 | 4.640 | 6.080 | 9.860 | 1659 | 2144 | 1.00 |
| Naldi L 2014 | 3.790 | 2.100 | 0.544 | 2.280 | 3.430 | 4.910 | 8.700 | 1068 | 802 | 1.00 |
| Al-Mutairi N 2014 | 5.600 | 2.090 | 2.310 | 4.080 | 5.290 | 6.780 | 10.400 | 2135 | 2097 | 1.00 |
| Jensen P 2013 | 4.920 | 2.110 | 1.670 | 3.460 | 4.610 | 6.040 | 9.940 | 1621 | 1784 | 1.00 |

**Figure S16.4:** Leave-one-out sensitivity analysis for between-study heterogeneity (τ) in PASI

**Table S16.4:** Leave-one-out sensitivity analysis of between-study heterogeneity (τ) for PASI after sequential exclusion of individual studies

| Study removed | τ (mean) | SD | 2.5% CrI | 25% | 50% | 75% | 97.5% CrI | Bulk ESS | Tail ESS | Rhat |
| --- | --- | --- | --- | --- | --- | --- | --- | --- | --- | --- |
| Primary analysis | 2.770 | 1.480 | 0.965 | 1.770 | 2.460 | 3.480 | 6.650 | 1843 | 2712 | 1.00 |
| Ismail AMA2023 (study 1) | 2.940 | 1.710 | 0.742 | 1.730 | 2.530 | 3.720 | 7.370 | 1372 | 2151 | 1.00 |
| Ismail AMA2023 (study 2) | 3.440 | 1.890 | 1.070 | 2.080 | 2.980 | 4.340 | 8.330 | 1356 | 2355 | 1.00 |
| Leite BF 2022 | 2.540 | 1.700 | 0.322 | 1.370 | 2.150 | 3.290 | 7.100 | 1122 | 1178 | 1.00 |
| Naldi L 2014 | 2.900 | 1.780 | 0.748 | 1.660 | 2.440 | 3.720 | 7.700 | 1589 | 1970 | 1.00 |
| Jensen P 2013 | 2.590 | 1.700 | 0.411 | 1.370 | 2.190 | 3.340 | 7.040 | 1265 | 1319 | 1.00 |

**Appendix 17: Narrative summary of DLQI and safety outcomes**

**Table S17.1:** Narrative summary of DLQI and safety outcomes across included studies

| Study | Outcome | Intervention | Comparator | n (T/C) | Results in intervention group | Results in comparator group | Notes |
| --- | --- | --- | --- | --- | --- | --- | --- |
| Ismail et al., 2023 | DLQI | Low-calorie diet | Comparator/control | 30/30 | 12.93 ± 5.32 | 17.36 ± 4.90 | Lower DLQI scores indicate better dermatology-specific quality of life. |
| Leite et al., 2022 | Safety | Dietetic intervention | Comparator/control | 32/33 | 0 events | 3 events | Comparator-group events included medication changes for psoriatic arthritis (2/33); 1/33 did not return for follow-up. |
| Naldi et al., 2014 | Safety | Diet and physical exercise | Comparator/control | 151/152 | Hunger or increased fatigue: 17; constipation: 7 | Hunger or increazed fatigue: 4; constipation: 4 | Reported events were mainly mild tolerability-related symptoms. |

**Abbreviations:** DLQI, Dermatology Life Quality Index; T, treatment; C, comparator; SD, standard deviation.

**Note:** DLQI and safety outcomes were summarized narratively because these data were sparsely reported and not amenable to quantitative synthesis. DLQI values are presented as mean ± SD, as reported in the original study. Safety data are presented descriptively according to the original study reports.
